# Age‐Associated Senescence of Decidual Macrophages: A Key Mediator of Adverse Pregnancy Outcomes in Advanced Maternal Age

**DOI:** 10.1111/acel.70614

**Published:** 2026-07-01

**Authors:** Yujing Zhang, Yiming Zhang, Guangshun Gong, Zhijing Li, Wenjing Xiong, Xuhui Fang, Ning Lu, Di Wang, Yihui Li, Aihua Liao

**Affiliations:** ^1^ Institute of Reproductive Health, Center for Reproductive Medicine, Tongji Medical College Huazhong University of Science and Technology Wuhan China; ^2^ Laboratory of Animal Center Huazhong University of Science and Technology Wuhan China

**Keywords:** advanced maternal age, aging, decidual macrophages, mice, uterus

## Abstract

Senescence of immune cells can drive senescence of solid organs, and reversing immune cell senescence ameliorates organismal senescence phenotypes. As the second largest subset of decidual immune cells, macrophages play a critical role in pregnancy. However, whether decidual macrophages (DM) exhibit senescence in advanced maternal age (AMA) pregnancies (≥ 35 years old) and their involvement in AMA‐related adverse outcomes remain undefined. This study elucidates the phenotypic and functional characteristics of DM in AMA pregnancies through clinical samples, animal models, and in vitro experiments. It clarifies the mechanisms underlying DM aging and explores novel intervention strategies to reverse DM aging and improve pregnancy outcomes in AMA pregnancies. We found that the presence of DM senescence in AMA pregnancies, manifested by an increased proportion of the pro‐inflammatory phenotype M1, increased expression of senescence markers (P53 and SA‐β‐Gal), and decreased phagocytic capacity—all associated with the development of adverse outcomes in AMA. The mechanistic basis of DM senescence involves two key pathways: on the one hand, low expression of forkhead box O3 causes DM senescence by downregulating mitophagy. On the other hand, elevated IL‐6 in the uterine microenvironment exacerbates senescence. Adoptive transfer of young mouse bone marrow‐derived macrophages significantly rescues embryo resorption rates and placental development in AMA pregnant mice. Overall, our study uncovers novel mechanisms and therapeutic strategies for AMA‐related adverse pregnancy outcomes through a new perspective of uterine macrophage senescence, providing new intervention ideas to improve adverse pregnancy outcomes in AMA.

## Graphical abstract

Decidual macrophages (DM) in advanced maternal age (AMA) exhibit FOXO3 deficiency‐driven senescence, further accelerated by elevated uterine IL‐6. This manifests as pro‐inflammatory polarization, increased senescence markers, reduced phagocytosis, and impaired trophoblast function. It is associated with an increased risk of adverse pregnancy outcomes in AMA pregnancies. Adoptive transfer of young bone marrow‐derived macrophages rescues AMA pregnancy outcomes, offering a potential therapeutic strategy.

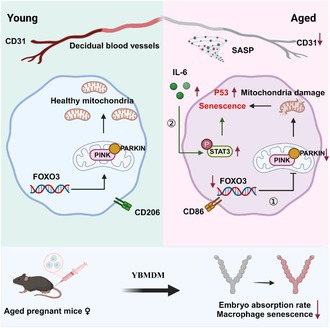

## 1 Introduction

Advanced maternal age (AMA) pregnancy, defined as pregnancy occurring in women aged 35 years or older (“Pregnancy at Age 35 Years or Older: ACOG Obstetric Care Consensus No. 11,” *Obstetrics and Gynecology* 2022), is characterized by a notable decline in fertility and an elevated risk of adverse pregnancy outcomes (Lean et al. 2017). These adverse outcomes encompass a spectrum of conditions, including repeated implantation failures, recurrent pregnancy loss, preterm birth, intrauterine fetal growth restriction, preeclampsia (PE), and gestational diabetes mellitus (Frick 2021; Mehari et al. 2020; Y. Y. Wang et al. 2021).

With advancing maternal age, the quality of oocytes undergoes a progressive deterioration, substantially increasing the risk of chromosomal abnormalities and miscarriages (Cavalcante et al. 2023; Pan et al. 2023). Although preimplantation genetic testing for aneuploidy (PGT‐A) facilitates the selection of euploid embryos, the cumulative live‐birth rate continues to decline with advancing maternal age‐from 54.8% in women under 35 years to 46.2% in women older than 42 years (Vitagliano et al. 2023). Moreover, even with young donor oocyte transfer, the incidence of adverse pregnancy outcomes, including implantation failure, PE, and fetal growth restriction, remains relatively high (Secomandi et al. 2022). These findings strongly suggest that, beyond oocyte‐related factors, uterine and placental factors play a crucial role in the increased risk of pregnancy outcomes associated with AMA. Uterine and placental senescence has thus been considered a significant contributor to age‐related pregnancy complications and fetal developmental abnormalities (Wu et al. 2023); however, research in this area remains limited.

Aging is characterized by irreversible cell cycle arrest, which is evidenced by multiple hallmarks, including telomere shortening, DNA damage, an elevation in β‐galactosidase (β‐Gal) activity, increased expression of cell cycle regulators such as P16, P21, and P53, as well as the upregulation of the senescence‐associated secretory phenotype (SASP) (Cohn et al. 2023; L. Zhang et al. 2022). Cellular senescence plays a central role in organismal aging, and targeted elimination of senescent cells has been shown to delay the onset and progression of age‐related diseases (Baker et al. 2011; L. Zhang et al. 2023). Consequently, identifying the specific senescent cell populations that most critically drive aging and serve as ideal therapeutic targets remains a pivotal area of investigation.

With advancing age, immune cells also undergo senescence, ultimately leading to functional impairment (Burton and Stolzing 2018). Senescent immune cells exhibit a diminished capacity to clear other senescent cells. As a result, these uneliminated senescent cells can accumulate within tissues, contributing to the development of various age‐related diseases, including neurodegenerative disorders, rheumatoid arthritis, cancer, cardiovascular diseases, and metabolic disorders (Z. Q. Liu et al. 2023; Santoro et al. 2021). Notably, immune aging can precede and even initiate the senescence of solid organs (Yousefzadeh et al. 2021). This finding suggests that immune cell senescence may represent a more crucial target for the prevention and treatment of age‐related diseases.

Woods et al. (2017) reported that in aged mice (12‐month‐old), the uterine decidualization at E11.5 was defective, accompanied by an elevated embryo resorption. Additionally, the proportion of placental macrophages and dendritic cells decreased, while the proportion of decidual natural killer (NK) cells remained unchanged. Decidual macrophages (DM), the second most abundant immune cell population in the decidua after NK cells, constitute 20%–30% of the total decidual leukocyte population (Faas and De Vos 2018; S. Liu et al. 2017). DM actively participate in crucial processes, including embryo implantation, placentation, fetal development, and parturition (Ning et al. 2016). Moreover, their abnormal polarization has been closely linked to the pregnancy complications, including recurrent pregnancy loss, preterm birth, and PE (X. Liu et al. 2022; Nagamatsu and Schust 2010; L. L. Wang et al. 2022).

Macrophages also play a significant role in the aging process. Minhas et al. (2021) found that, compared with young individuals (< 35 years), peripheral monocyte‐induced macrophages (MDMs) from elderly adults (> 65 years) exhibited increased production of prostaglandin E2 and its receptor prostaglandin E receptor 2 (EP2) following 20 h of culture. This was accompanied by reduced cognitive ability and decreased mitochondrial oxygen consumption. Notably, these effects were reversible in aged mice following treatment with EP2 inhibitors. Furthermore, studies have shown that aging induces a shift in macrophage polarization from the anti‐inflammatory “M2 type” to the pro‐inflammatory “M1 type”. This shift is accompanied by decreased expression of forkhead box O3 (FOXO3) and ultimately results in the degeneration of the intestinal nervous system, characterized by decreased neuronal density and prolonged intestinal transit time (Becker et al. 2018).

In conclusion, macrophages undergo senescence with age. Senescent macrophages can promote inflammation, cause tissue damage, and exacerbate disease progression. Notably, this ‘senescent phenotype’ is potentially reversible, offering a promising avenue for macrophage‐targeted therapeutic interventions. Thus, researching macrophage senescence holds substantial clinical and translational relevance. However, it remains unknown whether DM undergoe senescence during AMA pregnancies and how such changes may impact pregnancy outcomes. We hypothesize that DM acquire cellular senescence under conditions of AMA. DM senescence might be driven by intrinsic genetic alterations and extrinsic microenvironmental stimuli; senescent DM, in turn, compromise trophoblast function and normal placental development, ultimately increasing the risk of adverse gestational outcomes associated with AMA.

In this study, we uncover the senescence of DM in AMA pregnancy through clinical samples, murine models, and in vitro experiments. We characterize their phenotypic and functional features, clarify the underlying mechanisms of DM senescence, and explore novel intervention strategies to reverse DM senescence and improve pregnancy outcomes in AMA. Collectively, our work discovers previously unrecognized mechanisms contributing to adverse pregnancy outcomes in AMA, mediated by uterine macrophage senescence, and proposes innovative therapeutic approaches to mitigate these complications.

## 2 Methods

### 2.1 Mice

Female C57BL/6 (H‐2^b^) and male BALB/c (H‐2^d^) mice were employed and mated to construct an allogeneic pregnancy model (Huang et al. 2025). Cross‐mating mice with distinct genetic backgrounds can better recapitulate the authentic maternal‐fetal immune interplay in humans compared with syngeneic mating.

Young (3–5‐month‐old) or aged (10–12‐month‐old) female C57BL/6J mice were mated with male BALB/c mice at a 2:1 ratio. Vaginal plugs were checked at 9:00 a.m. the following morning, and the day of the detection was recorded as embryonic Day 0.5 (E0.5). All mice were provided with food and water freely, maintained under a 12‐h light/dark cycle at a room temperature of 20°C–25°C. All offspring were collected on the day of birth, followed by immediate body weight measurement and morphological photography. All animal procedures were approved by the Institutional Animal Care and Use Committee of Huazhong University of Science and Technology (Wuhan, China; IACUC number: 4723[2022]).

### 2.2 Clinical Sample Collection

Peripheral blood and tissue samples were collected from women in early pregnancy at the Department of Obstetrics and Gynecology, Hubei Provincial Maternal and Child Health Hospital (Wuhan, China). Clinical medical information was recorded (Table S1), and peripheral blood was collected in a heparin tube at room temperature, while decidual tissues were placed in sterile phosphate buffered saline (PBS) on ice. All samples were processed within 3 h of collection. All subjects were confirmed with viable intrauterine pregnancy by ultrasonography at 6–10 weeks of gestation and underwent electively surgical termination owing to unintended pregnancy. None of them had uterine anatomical abnormalities or underlying systemic diseases (e.g., heart disease or diabetes). Women with embryonic arrest or confirmed fetal abnormalities in the ongoing pregnancy were excluded from recruitment. This study was approved by the Clinical Trial Ethics Committee of Huazhong University of Science and Technology (Wuhan, China; CTEC number: S154[2021]).

### 2.3 Isolation of Decidual Macrophages

Leukocytes from decidual tissues were processed as previously described (L. L. Wang et al. 2022). Briefly, decidual tissue was minced into 1–3 mm^3^ fragments on ice with ophthalmic scissors. An appropriate amount of enzyme mixture containing 1 mg/mL hyaluronidase (Sigma, H3506), 150 μg/mL DNase I (Sigma, D2025), and 1 mg/mL collagenase (Biofeoxx, 209) was added. Tissues were digested in a 37°C water bath for 1 h. Decidual leukocytes were subsequently isolated by density gradient centrifugation using percoll (Biosharp, BS909). Cells at 25%/50% interface were carefully collected, wash twice with PBS, and centrifuged to pellet the cells. The cell pellet was resuspended in 2 mL erythrocyte lysis buffer, incubated on ice for 10 min, washed with PBS, and resuspended in PBS for flow cytometry (FCM).

### 2.4 Flow Cytometry and Cell Sorting

Cell suspensions were incubated with FCM surface antibody for 30 min at 4°C in the dark, then washed and resuspended with PBS. For intracellular staining, after surface antibody incubation, cells were incubated on ice for 20 min with Fixation/Permeabilization Kit (BD, 554714), washed, and resuspended before incubation with intracellular antibodies for 30 min. For intranuclear staining, after surface antibody incubation, cells were incubated on ice for 45 min using the Nucleoblast Membrane Breaking Kit (Biolegend, 424401), resuspended in Perm Buffer (Biolegend, 424401), stained with intranuclear antibody for 30 min, washed, and resuspended with perm buffer. For secretory cytokine antibodies, the cell pellets were resuspended in 1 mL of 1640 complete medium and stimulated with 2 μL Cell Stimulation Cocktail (ebioscience, 00‐4975‐03) for 4–6 h in a 37°C CO_2_ incubator. The surface and intracellular antibodies were then stained. Use flow cytometer (Agilent, NovoCyte) to detect as soon as possible. The list of fluorescent antibodies used is shown in Table S2.

DM were sorted by fluorescence‐activated cell sorting (FACS). The prepared decidual leukocyte suspensions were incubated with anti‐CD14 antibody (BioLegend, 308104) for 30 min at 4°C in the dark. Human DM sorting was performed using a high‐speed sorting flow cytometer (Beckman, CytoFLEX SRT), and sorted cells were collected into 1.5 mL Eppendorf tubes containing 0.5 mL 1640 complete medium.

### 2.5 RT–qPCR

Total RNA was extracted using TRIzol reagent, following the manufacturer's instructions. The concentration was measured using a microspectrophotometer (Thermo, Nanodrop2000), and each sample was tested 2–3 times, taking the average value. cDNA was extracted according to the instructions of the reverse transcription kit (Vazyme, R333). RT‐qPCR detection was performed on q225 (Kubo) using 2 × Taq Pro Universal SYBR qPCR Master Mix (Vazyme, q712). All data were normalized to β‐actin expression levels. Primer sequences are listed in Table S3.

### 2.6 Embryo Transfer

On day 0 (D0), female C57BL/6 mice were injected intraperitoneally with serum gonadotropin (8–10 IU/each) at 17:00. After 44–48 h, chorionic gonadotropin (8–10 IU/mouse) was administered intraperitoneally. Human tubal fluid was placed at 37°C in a CO_2_ incubator overnight to maintain a stable pH. On Day 3, spermatozoa were isolated from 3 to 5‐month‐old male BALB/c mice and underwent capacitation. Oocyte retrieval was routinely performed in young (3–5‐month‐old) and aged (10–12‐month‐old) female C57BL/6 mice. Embryos were washed following in vitro fertilization of spermatozoa and oocytes. The recipient females were housed with the vasectomy males the day before transfer and the tubes were transplanted the morning of the injection site. After the operation, they were placed in the insulated bed and waited for awakening before being placed in the mice chamber. The experimental groups were as follows: YE → Y group (*n* = 3): Embryos from young donors were transplanted to young recipient mice, YE → A group (*n* = 3): Embryos from young donors were transplanted to aged recipient mice, AE → Y group (*n* = 3): Embryos from aged mice were transplanted to young recipient mice. The embryos and placental tissues of the mice on E11.5 days were collected for experiments.

### 2.7 Experimental Treatments

Aged pregnant mice were generated by mating 10–12‐month‐old female C57BL/6 mice with 3–5‐month‐old male BALB/c mice. These mice were randomly assigned to four experimental groups, with intervention regimens tailored as follows: ① PBS group: On E6.5, mice received 100 μL PBS via both tail vein and intraperitoneal injection; an additional 100 μL PBS was administered via tail vein on E7.5. ② Bone marrow‐derived macrophages from young mice (YBMDM) group: On E6.5 and E7.5, mice were injected with 2 × 10^6^ YBMDM (resuspended in 100 μL of PBS) via the tail vein, respectively. ③ Anti‐interleukin‐6 receptor alpha antibody (αIL‐6R) group: On E6.5, mice received 100 μL of αIL‐6R solution (2 mg/mL in PBS) via intraperitoneal injection. ④ YBMDM+αIL‐6R group: On E6.5, mice were injected with 2 × 10^6^ YBMDMs (in 100 μL PBS) via tail vein and 100 μL αIL‐6R solution (2 mg/mL in PBS) via intraperitoneal injection; an additional 2 × 10^6^ YBMDMs (in 100 μL PBS) were administered via tail vein on E7.5. On E11.5, embryonic and placental tissues were harvested from mice across all experimental groups for subsequent analyses.

### 2.8 Statistical Analysis

Statistical data analysis was performed using GraphPad Prism 8.0 software. An unpaired *t*‐test or Mann–Whitney *U* test was used to compare the data between the two groups. For multigroup data comparison, one‐way ANOVA was used for normal data, and the Kruskal–Wallis test was used for non‐normal data. For multigroup data comparison involving two independent variables of maternal age and paternal age, two‐way ANOVA was used, followed by post hoc tests for intergroup comparison. Data were presented as the mean ± standard error of the mean (SEM). A *p*‐value of < 0.05 was considered statistically significant. Significance was indicated as follows: *p < 0.05, **p < 0.01, ***p < 0.001, ****p < 0.0001.

## 3 Results

### 3.1 Senescence of Decidual Macrophages Is Observed in Advanced Maternal Age Pregnancy

**FIGURE 1:**
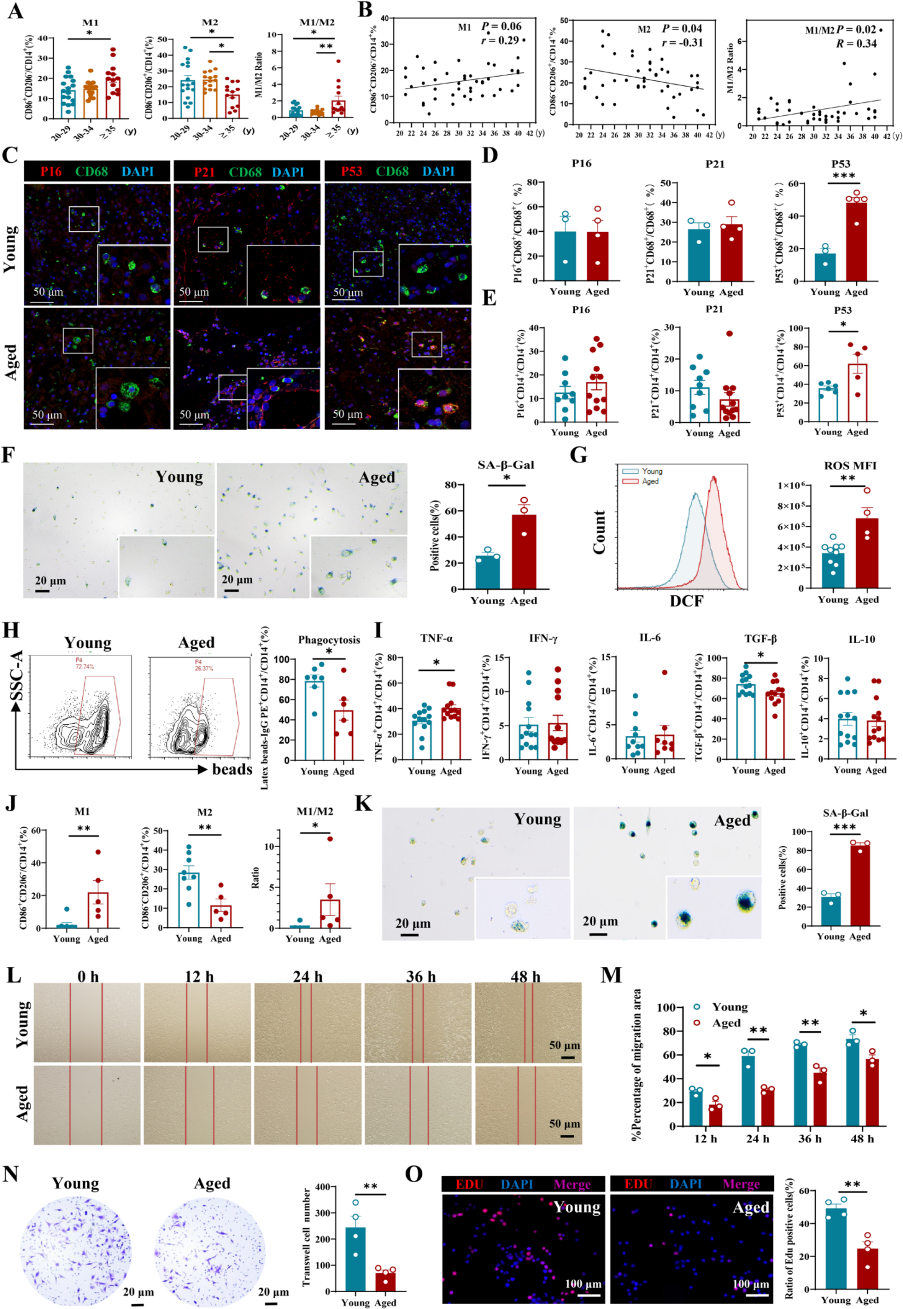
Decidual macrophages (DM) in early pregnancy of aged women exhibit senescence phenotypes compared to those of young women. (A) Flow cytometry (FCM) was employed to detect the proportions of M1 (CD14⁺CD86⁺CD206⁻) and M2 (CD14⁺CD86⁻CD206⁺) macrophages, as well as the M1/M2 ratio in DM of early pregnancy from women of different age groups (20–29 years group: *n* = 17, 30–34 years group: *n* = 15, ≥ 35 years group: *n* = 13). (B) Spearman correlation analysis was conducted to explore the relationship between the proportions of M1 and M2 macrophages, the M1/M2 ratio, and age in human early pregnancy (*n* = 45). (C, D) Immunofluorescence staining was used to examine the co‐localization of human early pregnancy DM (marked by CD68) with senescence markers (P16, P21, and P53) in both young and aged groups (Young group: *n* = 3, Aged group: *n* = 4). Data at each data point represent the mean value quantified from 3 to 5 randomly selected microscopic fields per tissue section. Scale bar = 50 μm. (E) The proportions of positive cells for senescence markers (P16: Young group, *n* = 9; Aged group, *n* = 12, P21: Young group, *n* = 9; Aged group, *n* = 12, and P53: Young group, *n* = 6; Aged group, *n* = 5.) in human early pregnancy DM were compared between the young and aged groups. (F) Representative images and quantitative analysis of positive cells from SA‐β‐Gal staining in human early pregnancy DM of the two groups, with a scale bar of 20 μm (*n* = 3). (G) Representative FCM images and cellular reactive oxygen species (ROS) levels in human early pregnancy DM of the young and aged groups (Young group: *n* = 9, Aged group: *n* = 4). (H) The phagocytic ability of DM between the two groups (Young group: *n* = 7, Aged group: *n* = 6). (I) Quantitative analysis of cytokines secretion (IL‐6, TNF‐α, IFN‐γ, TGF‐β, and IL‐10) in human early pregnancy DM from young and aged groups (*n* = 13). (J) FCM was utilized to detect the proportions of M1 (CD14⁺CD86⁺CD206⁻) and M2 (CD14⁺CD86⁻CD206⁺) macrophages, as well as the M1/M2 ratio, in early pregnancy monocyte‐derived macrophages (MDMs) from the young and aged groups (Young group: *n* = 8, Aged group: *n* = 5). (K) Representative images of SA‐β‐Gal staining in MDMs from early pregnancy in the young and aged groups (*n* = 3). Scale bar = 20 μm. (L–O) The effects of 24‐hour culture supernatants from MDMs of the young and aged groups on the migration (L, M), invasion (N), and proliferation (O) abilities of the trophoblastic cell line HTR‐8/SVneo were observed using light microscopy (*n* = 3–4). The scale bars were 50 and 20 μm, respectively. Young group: 20–29 years; Aged group: ≥ 35 years. DM, decidual macrophages; FCM, flow cytometry; IFN‐γ, interferon‐gamma; IL‐10, interleukin‐10; IL‐6, interleukin‐6; MDMs, monocyte‐derived macrophages; TGF‐β, transforming growth factor‐β; TNF‐α, tumor necrosis factor‐α. All data were presented as the mean ± standard error of the mean (SEM). The independent samples *t*‐test was applied for comparisons between the two groups. *p < 0.05, **p < 0.01, ***p < 0.001.

To explore age‐related differences in DM polarization during early pregnancy, FCM was utilized for phenotypic analysis. Compared with the 20–29‐year‐old group, the ≥ 35‐year‐old group (AMA group) exhibited a significantly higher proportion of M1 macrophages (CD14^+^CD86^+^CD206^−^), a lower proportion of M2 macrophages (CD14^+^CD86^−^CD206^+^), and an elevated M1/M2 ratio (Figure 1A). Meanwhile, compared to the 30–34‐year‐old group, the AMA group also showed a marked reduction in the proportion of M2 macrophages and an increase in the M1/M2 ratio. However, no statistically significant differences were found between the 30–34‐year‐old and 20–29‐year‐old groups (Figure 1A). Correlation analysis further revealed a negative association between maternal age and the proportion of M2 macrophages in early pregnancy, while the M1/M2 ratio positively correlated with age (Figure 1B). Based on these findings, subsequent analyses were conducted using the following two subgroups: young group (20–29‐year‐old) and aged group (≥ 35‐year‐old).

To compare the senescence phenotype of DM during early pregnancy between young and aged groups, immunofluorescence and FCM were used to detect the senescence markers, including P16, P21, and P53. Immunofluorescence analysis showed a significantly higher proportion of cells co‐expressing P53 (a senescence marker) and CD68 (a macrophage marker) in the aged group compared with the young group (Figure 1C,D). Similarly, FCM analysis showed a significantly increased proportion of P53^+^ DM in the aged group (Figure 1E). Furthermore, human DM isolated by fluorescence‐activated cell sorting (FACS) exhibited a significant increase in SA‐β‐Gal expression in the aged group (Figure 1F). Meanwhile, DM in the aged group showed significantly higher levels of reactive oxygen species (ROS) and reduced phagocytic capacity (Figure 1G,H). The production level of tumor necrosis factor‐α (TNF‐α) (a pro‐inflammatory cytokine) was elevated, whereas the level of transforming growth factor‐β (TGF‐β) (an anti‐inflammatory cytokine) was decreased in the aged group compared to the young group (Figure 1I). Collectively, these findings indicate that human DM from AMA pregnancies exhibit distinct senescent‐associated phenotypes.

DM are partially derived from the differentiation of peripheral monocytes (Sun et al. 2021). To further compare the phenotypic and functional alterations, we compared human MDMs in early pregnancy between young and aged groups. FCM results showed an increased proportion of M1 macrophages, a decreased proportion of M2 macrophages, and an increased ratio of M1/M2 in the aged group compared to the young group (Figure 1J). SA‐β‐Gal staining further confirmed a significant increase in MDMs of the aged group (Figure 1K), consistent with the findings observed in DM. To assess the functional impact, culture supernatants from MDMs were collected and used to co‐culture with the trophoblast cell line HTR‐8/SVneo cells for 24 h. We found that the culture supernatants in the aged group inhibited the migration (Figure 1L,M), invasion (Figure 1N), and proliferation (Figure 1O) of HTR‐8/SVneo cells compared to the young group, indicating a potential link to adverse pregnancy outcomes in AMA. Collectively, these findings demonstrate that MDMs' senescence also exists in AMA pregnancies, which may contribute to the compromised trophoblast cell functions.

### 3.2 Adverse Pregnancy Outcomes Are Increased in Advanced Maternal Aged Mice

**FIGURE 2:**
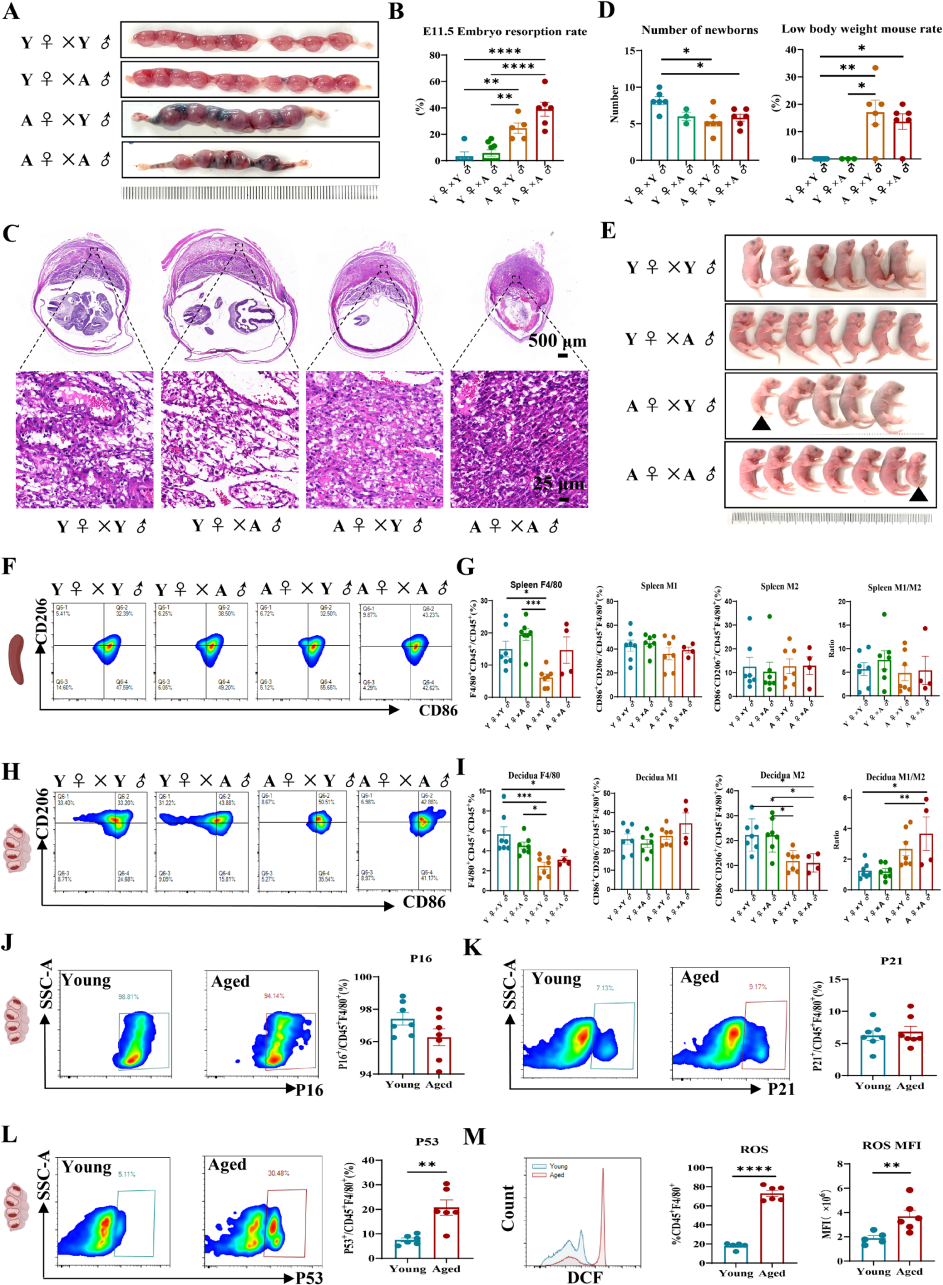
Increased incidence of adverse pregnancy outcomes in aged female mice associated with decidual macrophage senescence. (A, B) Representative images and statistical plots of embryo absorption rates at E11.5 days in different mouse groups (Y ♀ × Y ♂ group: *n* = 5; Y ♀ × A ♂ group: *n* = 9; A ♀ × Y ♂ group: *n* = 5; A ♀ × A ♂ group: *n* = 6). (C) Representative placental HE staining images at E11.5, with the magnified region showing the decidua layer, scale bar = 500 μm. (D, E) Statistical plots of newborn numbers and low‐birth‐weight mouse rates, along with representative images of mouse litters on birth day across groups (Y ♀ × Y ♂ group: *n* = 6; Y ♀ × A ♂ group: *n* = 3; A ♀ × Y ♂ group: *n* = 6; A ♀ × A ♂ group: *n* = 6). (F–I) FCM analysis of spleen (F, G) and decidua (H, I) total macrophage proportions (CD45^+^F4/80^+^), M1 (CD45^+^F4/80^+^CD86^+^CD206^−^) and M2 (CD45^+^F4/80^+^CD86^−^CD206^+^) macrophage subsets, and M1/M2 ratio at E11.5 days in different mouse groups (Y ♀ × Y ♂ group: *n* = 7; Y ♀ × A ♂ group: *n* = 7; A ♀ × Y ♂ group: *n* = 7; A ♀ × A ♂ group: *n* = 4). (J–L) Quantification of DM senescence marker‐positive cells (P16, P21, and P53) at E11.5 days between the aged and young mouse groups (*n* = 6–7). (M) FCM detection of reactive oxygen species (ROS) accumulation in DM at E11.5 days in the aged and young mouse groups (Young group: *n* = 5, Aged group: *n* = 6). Young group: C57BL/6♀ (3–5‐month‐old) × BALB/c ♂ (3–5‐month‐old), Aged group: C57BL/6 ♀ (10–12‐month‐old) × BALB/c ♂ (3–5‐month‐old). Y ♀ × Y ♂ group: C57BL/6 ♀ (3–5‐month‐old) × BALB/c ♂ (3–5‐month‐old), Y ♀ × A ♂ group: C57BL/6♀ (3–5‐month‐old) × BALB/c ♂ (10–12‐month‐old), A ♀ × Y ♂ group: C57BL/6 ♀ (10–12‐month‐old) × BALB/c ♂ (3–5‐month‐old), A ♀ × A ♂ group: C57BL/6 ♀ (10–12‐month‐old) × BALB/c ♂ (10–12‐month‐old). DM, decidual macrophages; FCM, flow cytometry; M, months. Data were presented as the mean ± SEM. Statistical analyses: Independent sample *t*‐test for two‐group comparisons; one‐way ANOVA for multi‐group comparisons; Figure 2B,D,G,I by using two‐way ANOVA. *p < 0.05, **p < 0.01, ***p < 0.001, ****p < 0.0001.

As previously reported (Ratto et al. 2019), 3–5‐month‐old mice were designated as the young group, while 10–12‐month‐old mice were designated as the aged group. To investigate gender‐specific effects on AMA‐related pregnancy complications, mice were divided into four groups: ① Y ♀ × Y ♂ group: C57BL/6 ♀ (3–5‐month‐old) × BALB/c ♂ (3–5‐month‐old), ② Y ♀ × A ♂ group: C57BL/6♀ (3–5‐month‐old) × BALB/c ♂ (10–12‐month‐old), ③ A ♀ × Y ♂ group: C57BL/6 ♀ (10–12‐month‐old) × BALB/c ♂ (3–5‐month‐old), ④ A ♀ × A ♂ group: C57BL/6 ♀ (10–12‐month‐old) × BALB/c ♂ (10–12‐month‐old). Embryos and placentas were collected at embryonic day (E)11.5, with the day of vaginal plug detection defined as E0.5. Increased embryo resorption rates in aged females—but not aged males—were observed (Figure 2A,B). HE staining of placentas revealed reduced placental area, dense decidual layers, decreased vascularization, and diminished labyrinth layer area in A ♀ × Y ♂ and A ♀ × A ♂ groups, potentially impairing fetal development (Figure 2C). Post‐delivery (on the day of birth) analyses showed that age‐related decreases in the number of viable fetuses and the increases in low‐birth‐weight fetuses (defined as ≥ 20% below average birth weight) were female‐dependent (Figure 2D,E). Collectively, our results demonstrate that the increased risk of age‐related adverse pregnancy outcomes in mice is associated with female mice, rather than male mice. A limitation of the present study is that the sex of newborn pups was not recorded or stratified in the analyses. Given established sex‐specific differences in fetal growth trajectories, placental function, and immune tolerance at the maternal–fetal interface, unmeasured sex effects may represent a potential confounder in the interpretation of our newborn weight data. Future studies incorporating fetal sex as a covariate will be essential to delineate any sexually dimorphic contributions to AMA‐associated adverse outcomes.

### 3.3 Senescence of Decidual Macrophages Also Exists in Advanced Maternal Aged Mice

To further confirm our findings in human DM at AMA, we detected macrophage subsets from spleen and decidua at E11.5 using FCM. In the spleen, the proportion of total macrophages (F4/80^+^) was significantly reduced in the A♀ × Y♂ group compared with the Y♀ × Y♂ group and the Y♀ × A♂ group, while no statistical difference was observed in that of polarized macrophage subsets (Figure 2F,G). In the decidua, aged female mice (A♀ × Y♂ and A♀ × A♂ groups) exhibited markedly low proportions of total DM (F4/80^+^) and M2‐type macrophages (CD86^−^CD206^+^), along with elevated M1/M2 ratios compared to young female mice (Y♀ × Y♂ and Y♀ × A♂ groups), independent of male age (Figure 2H,I).

**FIGURE 3:**
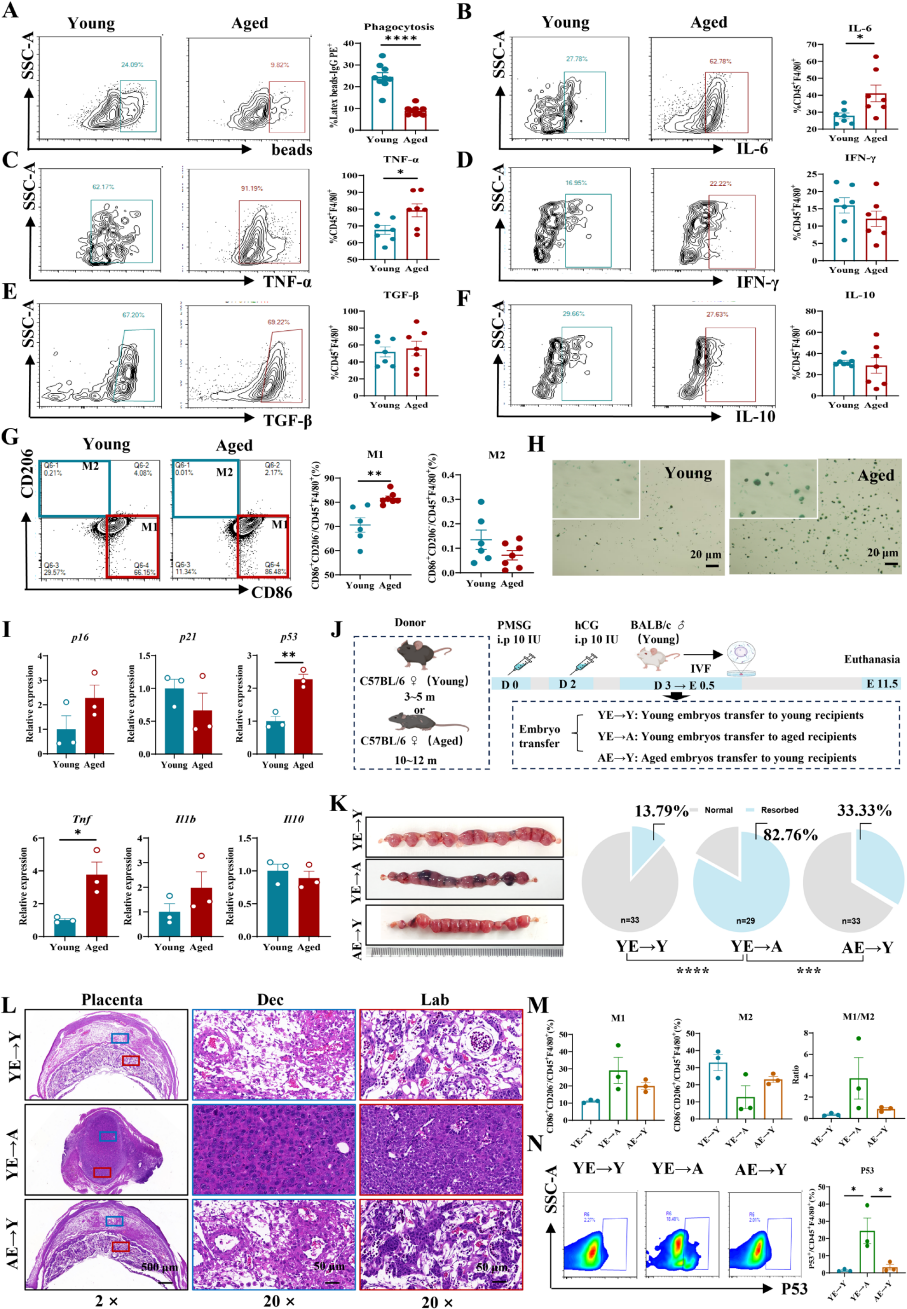
Alterations in decidual macrophages in aged mice are associated with adverse pregnancy outcomes. (A–F) FCM analysis of phagocytic (*n* = 9) activity of DM (A) and secretion levels of cytokines (B–F), including IL‐6, TNF‐α, IFN‐γ, TGF‐β, and IL‐10 (*n* = 7), in young and aged mouse groups at E11.5. Young group: C57BL/6♀ (3–5‐month‐old) × BALB/c ♂ (3–5‐month‐old); Aged group: C57BL/6 ♀ (10–12‐month‐old) × BALB/c ♂ (3–5‐month‐old). (G) FCM quantification of M1 (CD45^+^F4/80^+^CD86^+^CD206^−^) and M2 (CD45^+^F4/80^+^CD86^−^CD206^+^) macrophage subsets in BMDM from young (3–5‐month‐old; *n* = 6) and aged (10–12‐month‐old; *n* = 7) mice. (H) Representative images of SA‐β‐Gal staining of BMDM from young and aged mice. Scale bar = 20 μm. (I) RT‐qPCR analysis of mRNA expression levels of senescence markers (*p16*, *p21*, and *p53*) and cytokines (*Tnf*, *Il1b*, and *Il10*) in BMDM from young and aged mice (*n* = 3). (J) Schematic flowchart of the embryo transfer mouse model construction. YE → Y group (*n* = 3): Embryos from young (3–5‐month‐old) donors transplanted to young recipient mice; YE → A group (*n* = 3): Young donor embryos transplanted to aged (10–12‐month‐old) recipients; AE → Y group (*n* = 3): Aged donor embryos transplanted to young recipients. (K, L) Comparison of embryo absorption rate (*n* = 3) and placental HE staining at E11.5 across groups. Scale bars = 500 and 50 μm, respectively. The blue and red frames indicate magnified regions of the decidua (Dec) and labyrinth (Lab) layers, respectively. (M, N) FCM analysis of M1 and M2 macrophage proportion, M1/M2 ratio, and P53‐positive cell percentage in DM at E11.5 across different groups (*n* = 3). BMDM, bone marrow‐derived macrophages; DM, decidual macrophages; FCM, flow cytometry; IFN‐γ, interferon‐gamma; IL‐10, interleukin‐10; IL‐6, interleukin‐6; TGF‐β, transforming growth factor‐β; TNF‐α, tumor necrosis factor‐α. Data were presented as the mean ± SEM. Independent sample *t*‐test was used to compare two groups. Multiple sample rates were compared using Fisher's exact test. *p < 0.05, **p < 0.01, ****p < 0.0001.

Based on the above findings, mice were then divided into two groups for subsequent studies: ① Young group: C57BL/6 ♀ (3–5‐month‐old) × BALB/c ♂ (3–5‐month‐old), ② Aged group: C57BL/6 ♀ (10–12‐month‐old) × BALB/c ♂ (3–5‐month‐old). FCM analysis showed that the proportion of P53^+^ DM, the proportion of ROS, and the mean fluorescence intensity significantly increased in the aged group compared with the young group (Figure 2J–M). Moreover, DM in the aged group at E11.5 displayed reduced phagocytic capacity (Figure 3A), along with significantly increased production of pro‐inflammatory cytokines, including IL‐6 and TNF‐α (Figure 3B,C). By contrast, no significant differences were observed in the production of such cytokines as IFN‐γ, TGF‐β, and IL‐10 (Figure 3D–F). Collectively, these findings indicate that DM in AMA mice also exhibit senescent phenotypes and functional alterations, consistent with the results in humans.

Given that DM in mice are partially derived from bone marrow cells, we then used bone marrow‐derived macrophages (BMDM) from young and aged mice as cell models. FCM results showed an increased proportion of M1 polarized BMDM in the aged group compared with the young group (Figure 3G). Concurrently, SA‐β‐Gal expression was significantly elevated in BMDM from the aged group (Figure 3H). Real time Quantitative Polymerase Chain Reaction (RT‐qPCR) analysis further demonstrated that the mRNA expression levels of *p53* and *Tnf* were significantly upregulated in BMDM from the aged group compared with those from the young group (Figure 3I). Taken together, our results demonstrate that BMDM in AMA mice also exhibit senescent phenotypes.

To clarify whether age‐related effects on pregnancy outcomes are attributed to ovarian or uterine factors and their association with DM senescence, we established embryo transfer models (Figure 3J). Mouse embryos and placentas were collected at E11.5 for evaluation of fetal development. Transferring young embryos into aged recipients (YE → A) markedly increased embryo resorption rates, whereas transferring aged embryos into young recipients (AE → Y) had no effect (Figure 3K). Distinct from human reproductive physiology, over 85% of resorbed conceptuses from aged mice retain a normal euploid karyotype (Tao and Liu 2015), a critical interspecies difference accounting for the unaltered resorption rate in the AE → Y group. Our observations are concordant with Woods et al. (2017), who documented equivalent embryonic survival rates of 73% (AE → Y) and 83% (YE → Y). HE staining revealed reduced placental area, dense decidual and labyrinthine tissues, and decreased vascularization in YE → A mice at E11.5, suggesting impaired fetal development (Figure 3L). These results indicate that the elevated embryo resorption and placental dysplasia in advanced aged mice were related to the uterine microenvironment. FCM analysis showed that no significant differences in M1/M2 ratios among the groups. However, the proportion of P53^+^ DM (senescence cells) was markedly increased in YE → A mice at E11.5 (Figure 3M,N), which might be related to the increased embryo resorption.

### 3.4 FOXO3 as a Potential Key Driver of Decidual Macrophage Senescence

**FIGURE 4:**
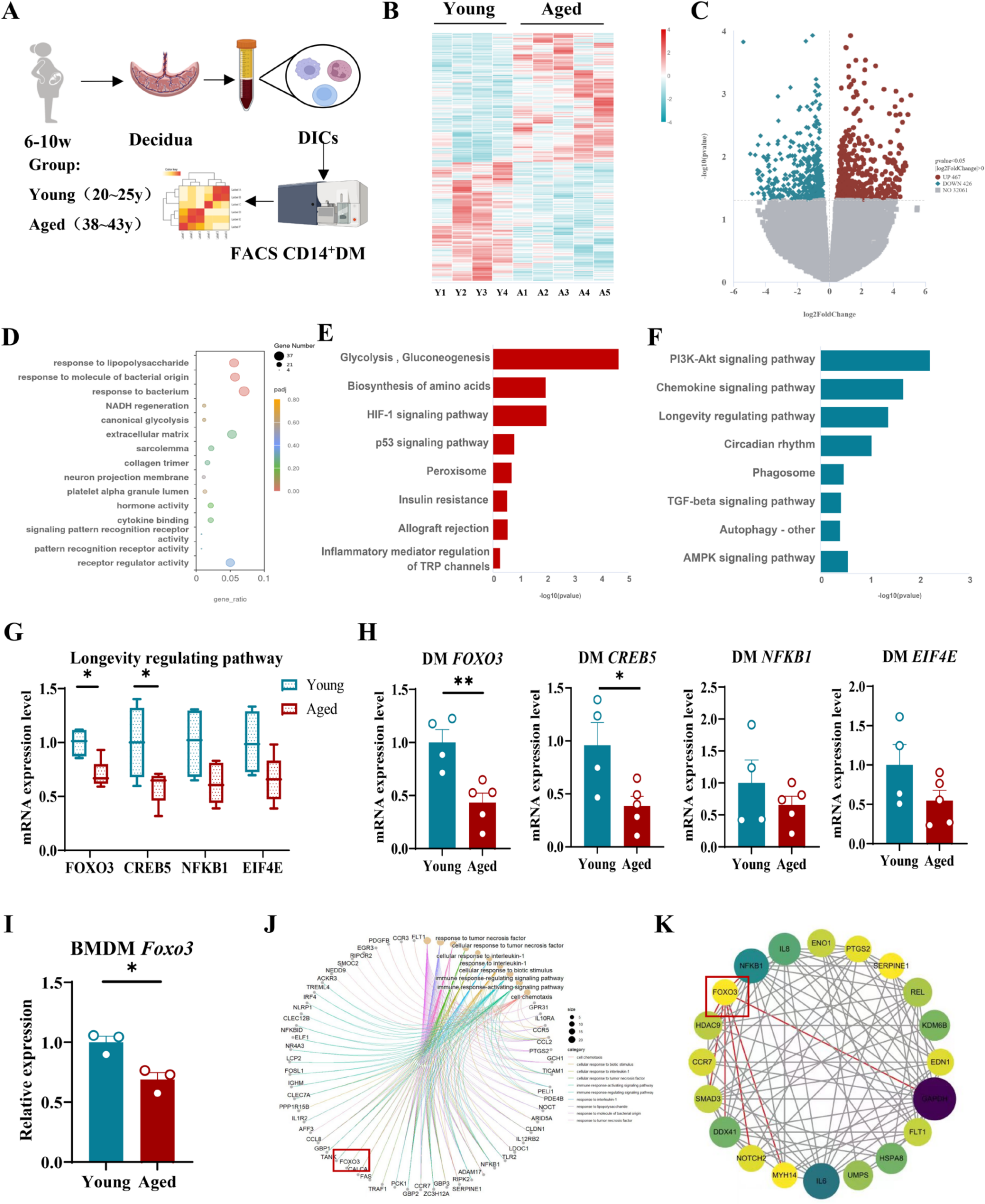
Identification of key genes driving decidual macrophage senescence. (A) Schematic illustration of the human DM sorting (Young group: *n* = 4, Aged group: *n* = 5). (B, C) Heat map (B) and volcano plot (C) depicting differentially expressed genes in human early pregnancy DM between young and aged groups. Red indicates upregulated genes, blue represents downregulated genes, and gray denotes genes with no significant change. (D–F) Kyoto Encyclopedia of Genes and Genomes (KEGG) pathway enrichment analysis of human DM during early pregnancy in young and aged groups. Upward pathways are marked in red, while downward pathways are shown in blue. (G) Visual analysis of the relative expression levels of genes enriched in the longevity‐related signaling pathways of human DM during early pregnancy in young and aged groups. (H) RT‐qPCR analysis of mRNA expression levels of genes (*FOXO3*, *CREB5*, *NFKB1*, and *EIF4E*) involved in longevity‐related signaling pathways in human early pregnancy DM from young and aged groups (Young group: *n* = 4, Aged group: *n* = 5). (I) RT‐qPCR quantification of *Foxo3* mRNA expression in BMDM from young and aged mouse groups (*n* = 3). (J) Network diagram illustrating the relationships among the top ten downregulated signaling pathways identified by Gene Ontology (GO) enrichment analysis. (K) Protein–protein interaction (PPI) network of DEGs in human early pregnancy DM between young and aged groups. Young group: 20–25 years; Aged group: 38–43 years. FACS: Fluorescence‐activated cell sorting; DM: Decidual macrophages. Data were presented as the mean ± SEM. Independent sample *t*‐test was used to compare two groups. *p < 0.05, **p < 0.001.

To explore key genes underlying DM senescence, we collected early pregnancy decidual tissues from young and aged groups, sorted human DM by FACS for RNA sequencing (Figure 4A). A total of 893 differentially expressed genes (DEGs) were identified, including 467 upregulated and 426 downregulated genes (Figure 4B,C). Gene ontology (GO) enrichment analysis showed that these DEGs were mainly enriched in “response to lipopolysaccharide”, “canonical glycolysis”, and “cytokine binding” pathways (Figure 4D). Kyoto encyclopedia of genes and genomes (KEGG) analysis revealed upregulated pathways in aged human DM, such as “glycolysis, gluconeogenesis”, “HIF‐1 signaling pathway”, and “insulin resistance” (Figure 4E), and downregulated pathways including “longevity regulating signaling pathway”, “TGF‐beta signaling pathway”, and “autophagy” (Figure 4F). Given the close association of the “longevity regulating signaling pathway” with cellular senescence, we focused on this pathway and observed reduced mRNA in the expression of *FOXO3* and *CREB5* in aged human DM based on transcriptomic profiling (Figure 4G). RT‐qPCR validation further confirmed significantly decrease expression of *FOXO3* and *CREB5* mRNA in aged human DM (Figure 4H). Consistent with human data, the expression of *Foxo3* mRNA was also significantly downregulated in aged mouse BMDM (Figure 4I). Meanwhile, GO enrichment analysis of the top 10 downregulated signaling pathways and protein–protein interaction network analysis of the top 20 genes both converged on FOXO3 (Figure 4J,K). It is known that the longevity‐associated gene FOXO3 plays a crucial role in regulating cell cycle arrest and apoptosis. Notably, reduced expression of FOXO3 has been closely linked to the aging process. Thus, we speculate that FOXO3 may act as a key regulator of DM senescence.

To investigate the regulatory role and underlying molecular mechanisms of FOXO3 knockdown in THP‐1‐derived macrophages' senescence, we established a stable FOXO3‐knockdown THP‐1 cell line. THP‐1 cells were transduced with lentiviral particles at a multiplicity of infection (MOI) of 10 in the presence of polybrene (5 μg/mL). At 3 days post‐infection, 1 μg/mL puromycin was added for selection, and the cells were cultured for 2 weeks until all uninfected cells were eliminated, yielding stable transfectants. Thereafter, maintenance was carried out using 0.5 μg/mL puromycin (Figure S1). Three FOXO3‐shRNA lentiviral constructs were selected and used to generate stable lines, designated as knockdown (KD) 1, KD2, and KD3, respectively. RT‐qPCR and western blotting (WB) analysis showed that only KD1 exhibited significantly decreased FOXO3 expression at both mRNA and protein levels. Consequently, KD1 was selected as the FOXO3‐knockdown THP‐1 cell line (hereafter referred to as the KD group), as validated in Figure S2.

**FIGURE 5:**
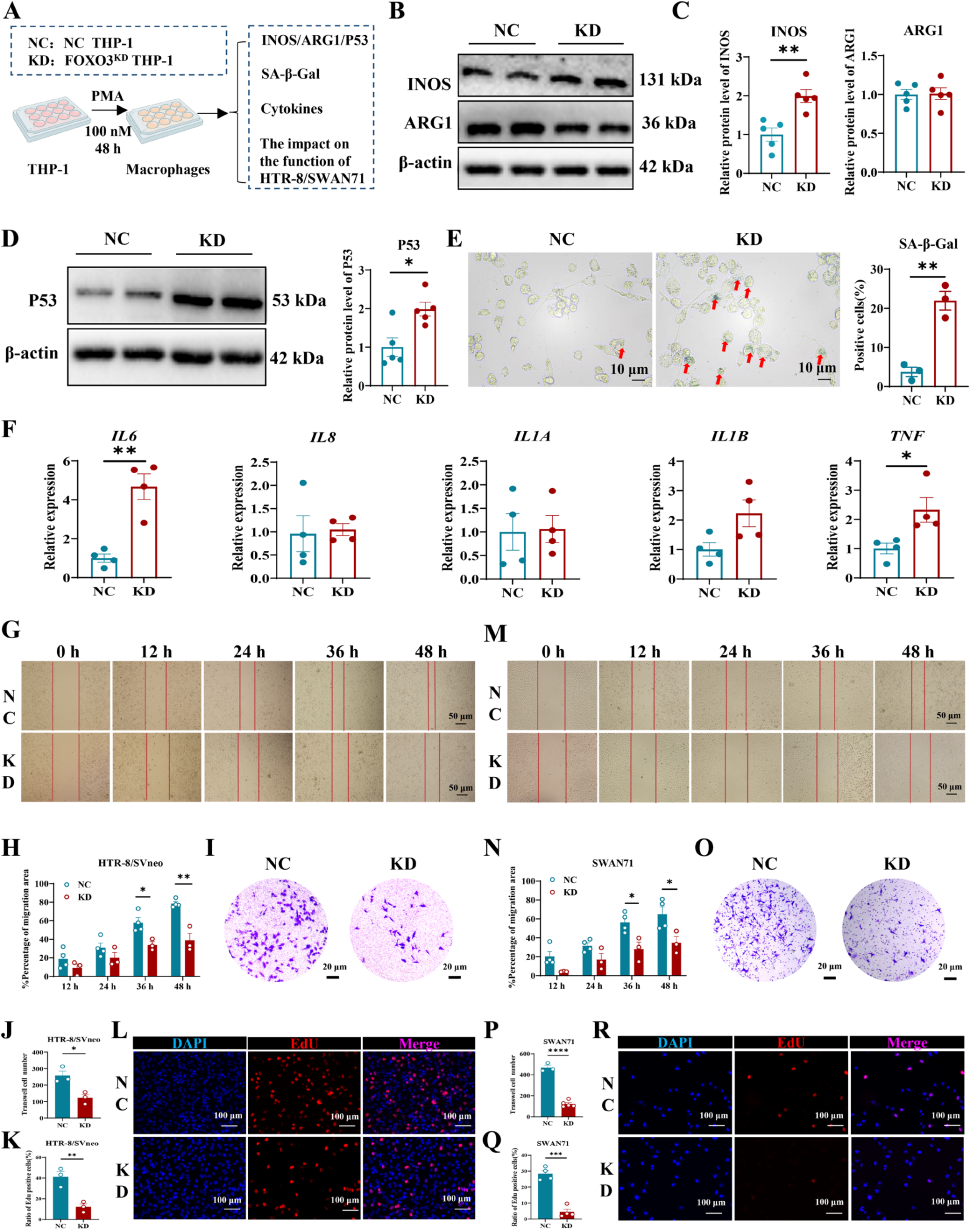
FOXO3 knockdown induces senescence in THP‐1 derived macrophages. (A) Schematic diagram of cell culture and experimental workflow. (B–D) Western blot (WB) analysis of protein expression levels of M1 macrophage marker iNOS (B, C), M2 marker ARG1 (B, C), and senescence marker P53 (D) in NC (negative control) and KD (FOXO3 knockdown) groups (*n* = 5). (E) Comparison of SA‐β‐Gal‐positive cell proportions between NC and KD groups (*n* = 3). Scale bar = 10 μm. (F) Comparison of mRNA expression levels of proinflammatory cytokines (*IL6*, *IL8*, *IL1A*, *IL1B*, and *TNF*) between NC and KD groups (*n* = 4). (G–L) Effect of 24‐hour conditioned supernatants from NC and KD groups on migration (G, H), invasion (I, J), and proliferation (K, L) of trophoblast cell line HTR‐8/SVneo. Scale bars = 100, 50, and 20 μm. (M–R) Effect of 24‐hour conditioned supernatants from NC and KD groups on migration (M, N), invasion (O, P), and proliferation (Q, R) of trophoblast cell line SWAN71(*n* = 3–5). Scale bars = 100, 50, and 20 μm. NC group: THP‐1 cells transduced with empty lentiviral vector; KD: THP‐1 cells transduced with FOXO3‐specific shRNA lentivirus. Data were presented as the mean ± SEM. Independent sample *t*‐test was used to compare two groups. *p < 0.05, **p < 0.01, ***p < 0.001, ****p < 0.0001.

THP‐1 cells from the negative control (NC) and KD groups with better growth status were differentiated into macrophages by treatment with 100 nM PMA for 48 h in vitro. Cells were collected for subsequent experiments (Figure 5A). WB analysis showed a significant increase in expression levels of the M1 macrophage marker INOS (Figure 5B,C) and senescence marker P53 protein (Figure 5D) in the KD group compared with the NC group. Additionally, the number of SA‐β‐Gal‐positive cells was significantly increased in the KD group versus the NC group (Figure 5E). RT‐qPCR results showed that the mRNA expression levels of *IL6* and *TNF* were significantly upregulated in the KD group, whereas *IL8*, *IL1A*, and *IL1B* levels remained unchanged (Figure 5F). These results demonstrate that *FOXO3* knockdown promotes senescence in THP‐1‐derived macrophages.

To further explore the effects of THP‐1‐derived macrophages in NC and KD groups on trophoblast functions, we used two trophoblast cell lines: HTR‐8/SVneo and SWAN71 cells. Culture supernatants from THP‐1‐derived macrophages were collected after 24 h and then co‐cultured with the two trophoblast cell lines, respectively. We found that 24‐h culture supernatant from the KD group significantly inhibits the migration, invasion, and proliferation of both HTR‐8/SVneo (Figure 5G–L) and SWAN71 (Figure 5M–R) cell lines, compared to the NC group.

### 3.5 FOXO3 Regulates Macrophage Senescence via Mitophagy

**FIGURE 6:**
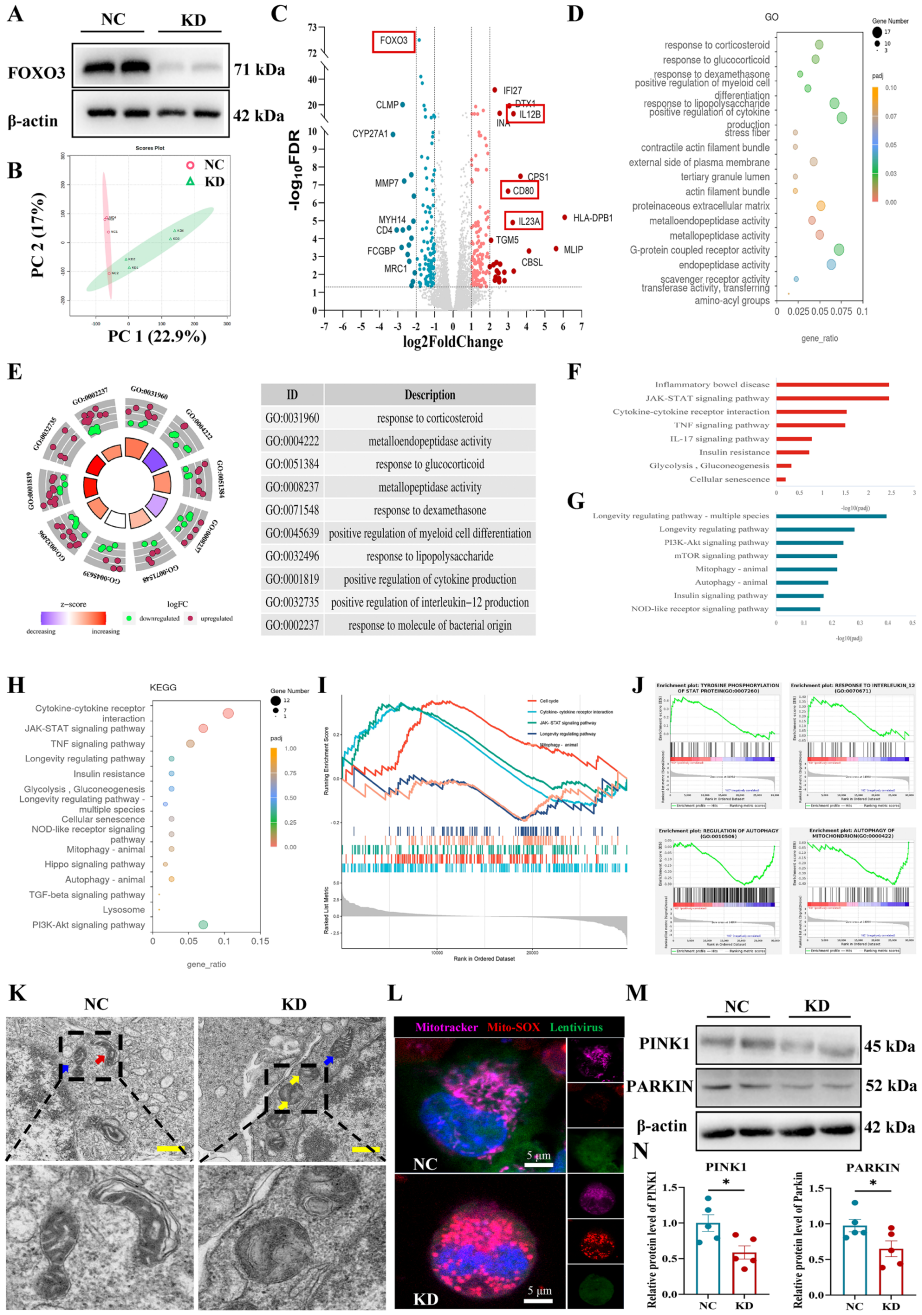
FOXO3 knockdown induces senescence in THP‐1‐derived macrophages via regulation of mitophagy. (A) WB analysis of FOXO3 protein expression levels in THP‐1 derived macrophages between the NC (negative control) and KD (FOXO3 knockdown) groups. (B) Principal component analysis (PCA) of gene expression profiles in THP‐1‐derived macrophages from NC and KD groups (*n* = 4). (C) Volcano plot of DEGs in THP‐1‐derived macrophages between the NC and KD groups. Red: Upregulated genes; blue: Downregulated genes; gray: Non‐significant changes. (D, E) Gene ontology (GO) functional enrichment analysis visualized as bubble plots (D) and circular plots (E) for THP‐1‐derived macrophages from the NC and KD groups. (F–H) Kyoto Encyclopedia of Genes and Genomes (KEGG) pathway enrichment analysis for THP‐1‐derived macrophages in NC and KD groups, with red indicating upregulated pathways and blue indicating downregulated pathways. (I, J) Gene set enrichment analysis (GSEA) of THP‐1 derived macrophages in NC and KD groups. (K) Transmission electron microscopy (TEM) images of mitochondria and mitophagy in THP‐1‐derived macrophages from the NC and KD groups. Blue arrows: Normal mitochondria; yellow arrows: Abnormal mitochondria; red arrows: Mitophagy. Scale bar = 0.5 μm. (L) Confocal microscopy analysis of mitochondria morphology (Mito‐tracker staining) and mitochondrial ROS (Mito‐SOX staining) in NC and KD groups. Scale bar = 5 μm. (M, N) WB analysis of PINK1 and PARKIN protein expression levels in THP‐1‐derived macrophages from the NC and KD groups (*n* = 5). NC group: THP‐1 cells transduced with empty lentiviral vector; KD: THP‐1 cells transduced with FOXO3‐specific shRNA lentivirus. Data were presented as the mean ± SEM. Independent samples *t*‐test was used to compare two groups. *p < 0.05.

To explore the underlying mechanisms of senescence in *FOXO3*‐knockdown THP‐1‐derived macrophages, RNA sequencing was performed on cells from the NC and KD groups (Figure 6A). Transcriptome analysis identified 157 upregulated and 122 downregulated genes between the two groups (Figure 6B,C). GO enrichment results showed that DEGs were mainly associated with “response to glucocorticoid”, “response to lipopolysaccharide”, and “positive regulation of cytokine production” (Figure 6D). GO circle plot analysis further highlighted the upregulation of pathways including “response to lipopolysaccharides”, “positive regulation of cytokine production”, and “positive regulation of interleukin‐12 production” in the KD group (Figure 6E). KEGG pathway enrichment analysis showed that DEGs were mainly enriched in “cytokine‐cytokine receptor interaction”, “cellular senescence”, and “mitophagy‐animal” pathways (Figure 6F–H). Among these, “cytokine‐cytokine receptor interaction”, “JAK‐STAT signaling pathway”, “TNF signaling pathway”, and “cellular senescence” were upregulated in the KD group (Figure 6F), whereas the “longevity regulating pathway” and “mitophagy‐animal” signaling pathway were downregulated (Figure 6G). Gene set enrichment analysis (GSEA) based on KEGG and GO datasets confirmed upregulation of “cell cycle”, “JAK–STAT signaling pathway”, “cytokine‐cytokine receptor interaction”, and “response to interleukin‐12” in the KD group, while downregulation of “longevity regulating pathway”, “mitophagy‐animal”, and “regulation of autophagy” (Figure 6I,J).

Mitophagy is a conserved longevity mechanism that maintains cellular energy homeostasis and eliminates damaged mitochondria to prevent oxidative stress, thereby preserving cellular integrity (Picca et al. 2023). FOXO3 has been reported to regulate mitophagy by transcriptional regulation of downstream PINK1 (Audesse et al. 2019). To determine whether FOXO3 modulates macrophage senescence via mitophagy, we employed transmission electron microscopy (TEM), confocal microscopy, and WB. TEM analysis showed that, compared with the NC group, the KD group exhibited fewer morphologically normal mitochondria, increased mitochondrial swelling and rounding, disrupted or absent cristae, and impaired mitophagy (Figure 6K). Confocal microscopy corroborated these findings, showing loss of elongated, rod‐shaped mitochondria (replaced by punctate structures) and significantly increased Mito‐SOX fluorescence (a marker of mitochondrial ROS) in the KD group (Figure 6L). Moreover, WB analysis further demonstrated reduced expression of PINK1 and PARKIN protein in the KD group (Figure 6M,N). Collectively, these findings demonstrate that FOXO3 depletion suppresses the PINK1/PARKIN mitophagy pathway, exacerbates mitochondrial damage, elevates mitochondrial ROS production, and promotes macrophage senescence.

### 3.6 Increased IL‐6 in the Uterine Microenvironment Drives Decidual Macrophage Senescence

**FIGURE 7:**
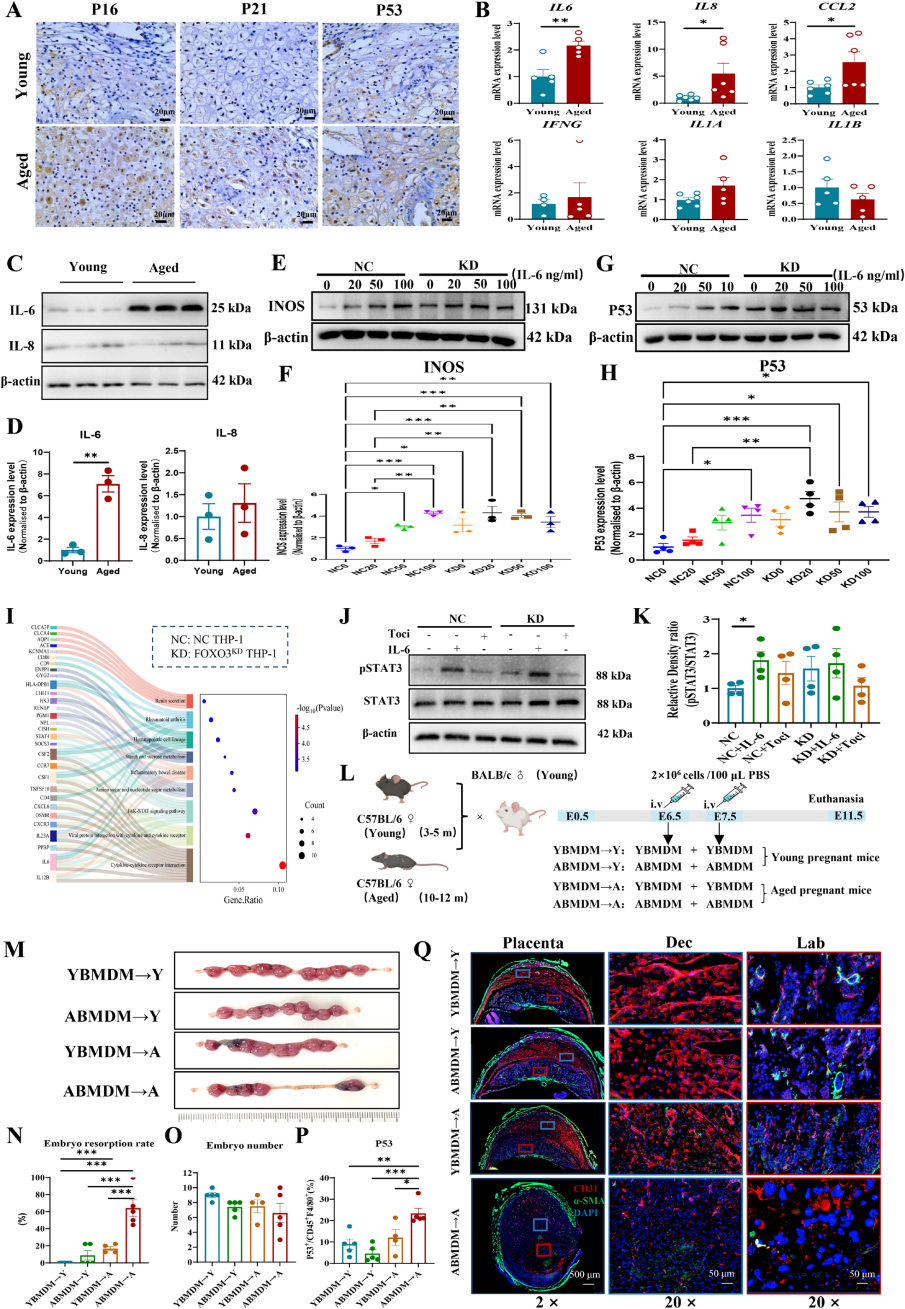
Differences in the uterine microenvironment between young and advanced maternal age. (A) Immunohistochemical staining of senescence markers (P16, P21, and P53) in human early pregnancy decidual tissues from young (20–29 years old) and aged (≥ 35 years old) groups. (B) RT‐qPCR analysis of SASP (*IL6*, *IL8*, *CCL2*, *IFNG*, *IL1A*, *IL1B*) mRNA expression levels in human early pregnancy decidual tissues from young and aged groups (*n* = 4–6). (C, D) WB analysis of IL‐6 and IL‐8 protein expression levels in decidual tissues from young and aged groups. (E–H) WB analysis of INOS (E, F) and P53 (G, H) protein expression levels in THP‐1‐derived macrophages from NC and KD groups (NC group: THP‐1 cells transduced with empty lentiviral vector; KD: THP‐1 cells transduced with FOXO3‐specific shRNA lentivirus) (*n* = 3). (I) KEGG pathway bubble plot from RNA sequencing of THP‐1‐derived macrophages in NC and KD groups. (J, K) WB analysis of STAT3 and p‐STAT3 protein expression in THP‐1‐derived macrophages in NC and KD groups (*n* = 4). (L) Schematic diagram of mouse model construction and grouping. YBMDM → Y group: Young BMDM (3–5‐month‐old donor) transferred to young pregnant recipients (3–5‐month‐old C57BL/6 ♂ × 3–5‐month‐old BALB/c ♂); ABMDM → Y group: Aged BMDM (10–12‐month‐old donor) transferred to young pregnant recipients; YBMDM → A group: Young BMDM transferred to aged pregnant recipients (10–12‐month‐old C57BL/6 ♂ × 3–5‐month‐old BALB/c ♂); ABMDM → A group: Aged BMDM transferred to aged pregnant recipients; All transfers were given twice via tail vein at E6.5 and E7.5, respectively. (M–O) Comparison of embryo absorption rate (M, N) and embryo numbers (M, O) at E11.5 across mouse groups (YBMDM → Y group: *n* = 5; ABMDM → Y group: *n* = 5; YBMDM → A group: *n* = 4; ABMDM → A group: *n* = 5). (P) Flow cytometry (FCM) analysis of P53^+^ cell proportions in DM at E11.5 across mouse groups (YBMDM → Y group: *n* = 5; ABMDM → Y group: *n* = 5; YBMDM → A group: *n* = 4; ABMDM → A group: *n* = 5). (Q) Representative immunofluorescence images of CD31 (red; labeling vascular endothelial cells) and α‐SMA (green; labeling smooth muscle cells) staining in placenta at E11.5. Magnified regions highlight the decidual layer (Dec, blue frame) and labyrinth (Lab, red frame) layers. Scale bars = 500 and 50 μm. BMDM, bone marrow‐derived macrophages; Dec, decidua layer; DM, decidual macrophages; Lab, labyrinthine layer; toci, tocilizumab, IL‐6 receptor blocker. Data were presented as the mean ± SEM. Statistical analyses: Independent sample *t*‐test for two‐group comparisons; one‐way ANOVA for multi‐group comparisons. *p < 0.05, **p < 0.01, ***p < 0.001.

It is known that genetic and environmental interactions drive aging and chronic inflammation, which in turn underlie various age‐related diseases (Campisi et al. 2019). While the previous sections focused on the gene‐regulatory mechanisms of macrophage senescence, we next discuss the influence of environmental factors on DM senescence. Immunohistochemical staining of human early pregnancy decidual tissues from young (20–29 years old) and aged (≥ 35 years old) individuals showed significantly higher expression of senescence markers including P16, P21, and P53, in the aged group (Figure 7A). RT‐qPCR results showed markedly increased mRNA levels of SASP‐related cytokines, including *IL6*, *IL8*, and *CCL2* in the decidua tissue of the aged group (Figure 7B), corroborated by increased IL‐6 protein expression confirmed by WB (Figure 7C,D). Collectively, these results indicate that the decidua microenvironment in the aged group exhibits an enhanced senescent phenotype, characterized by high inflammatory status.

To further validate whether IL‐6 acts as a key driver of DM senescence, THP‐1‐derived macrophages were treated with increasing concentrations of IL‐6 (0, 20, 50, or 100 ng/mL) for 48 h. In the NC group, the expression levels of INOS and P53 protein significantly increased in an IL‐6 dose‐dependent manner. Additionally, 20 ng/mL IL‐6 significantly upregulated the INOS and P53 expression in the KD group compared to NC (Figure 7E–H). Previous studies have reported that IL‐6/STAT3 pathway activation promotes M1 macrophage polarization in *Porphyromonas gingivalis* ‐induced apical periodontitis (X. Chen et al. 2022). Based on this, we speculated that IL‐6 may induce macrophage senescence through activating the STAT3 signaling pathway. KEGG pathway enrichment analysis revealed that IL‐6 was associated with several pathways, including “cytokine‐cytokine receptor interaction”, “JAK‐STAT signaling pathway” and “hematopoietic cell lineage” (Figure 7I). To further assess the potential FOXO3‐IL‐6‐STAT3 regulatory loop in macrophage senescence, THP‐1 cells were treated with IL‐6 or tocilizumab (an IL‐6 receptor antagonist). WB analysis showed a significant increase in phosphorylated STAT3 (pSTAT3) protein levels in the NC + IL‐6 group compared with the NC group (Figure 7J,K). The above results demonstrate that IL‐6 might induce macrophage senescence by activating pSTAT3 signaling.

### 3.7 Combined Genetic and Environmental Impacts on Pregnancy Outcomes via Decidual Macrophage Senescence in Mice

To determine how genetic and environmental factors influence the pregnancy outcomes via driving DM senescence, we performed in vivo experiments. Mice were grouped as follows: YBMDM → Y group, ABMDM → Y group, YBMDM → A group, and ABMDM → A group. Embryonic and placental tissues were collected at E11.5 for analysis (Figure 7L). The results showed that the embryo resorption rates were significantly decreased in the YBMDM → A and ABMDM → Y groups compared with the ABMDM → A group (Figure 7M,N), while the total number of embryos remained unchanged (Figure 7O). Similarly, FCM analysis showed a marked decrease in the proportion of P53^+^DM in the YBMDM → A and ABMDM → Y groups compared with the ABMDM → A group (Figure 7P), with no significant differences observed in polarized macrophage subsets (Figure S3A–D). DM originate from circulating monocytes and uterine‐resident macrophages (Sun et al. 2021). Hence, post‐transfer macrophage polarization is collectively shaped by transferred BMDM, endogenous resident macrophages, and the local decidual microenvironment. Histology and immunofluorescence (red: CD31 for marking vascular endothelium; green: α‐SMA for marking smooth muscle) showed that the placentas from the YBMDM → A and ABMDM → Y groups had sparser decidual layers, increased labyrinthine area, and more large‐lumen vessels compared with the ABMDM → A group (Figure S4). Also, CD31 expression was increased in both decidual and labyrinthine layers (Figure 7Q). Collectively, these findings indicate that genetic and environmental factors jointly influence the pregnancy outcomes in AMA mice via driving DM senescence.

### 3.8 Adoptive Transfer of Young BMDM Could Improve the Pregnancy Outcomes in Aged Mice

**FIGURE 8:**
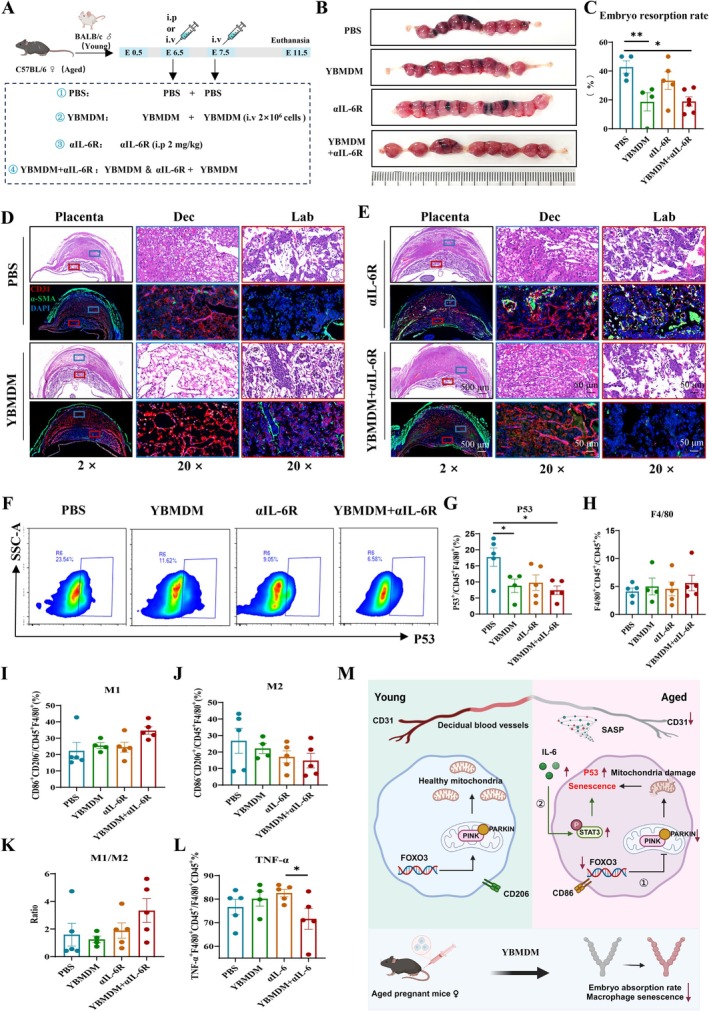
Adoptive transfer of young macrophages and αIL‐6R blockade improve pregnancy outcomes in advanced maternal age. (A) Schematic diagram of mouse model construction and grouping. PBS group: Control; YBMDM group: Adoptive transfer of young BMDM; αIL‐6R group: Treatment with αIL‐6R (IL‐6 receptor antibody); YBMDM + αIL‐6R group: Transfer of Young mouse BMDM combined with αIL‐6R blockade. (B, C) Comparison of embryo resorption rate at E11.5 across mouse groups (PBS group: *n* = 4; YBMDM group: *n* = 4; αIL‐6R group: *n =* 5; YBMDM+αIL‐6R group: *n* = 6). (D, E) Representative placental HE staining and immunofluorescence images of CD31 (red) and α‐SMA (green) staining at E11.5, with magnified regions of the decidua (blue frame) and labyrinth (red frame) layers. Scale bars = 500 and 50 μm. (F, G) FCM analysis of P53^+^ cell proportions in DM at E11.5 (PBS group: *N* = 5; YBMDM group: *n* = 4; αIL‐6R group: *n =* 5; YBMDM+αIL‐6R group: *n* = 5). (H–K) FCM quantification of the proportions of DM (H), M1 (I) and M2 (J) macrophage subsets, and M1/M2 ratio (K) at E11.5 (PBS group: *n* = 5; YBMDM group: *n* = 4; αIL‐6R group: *n =* 5; YBMDM + αIL‐6R group: *n* = 5). (L) FCM detection of TNF‐α secretion by DM at E11.5 (*n* = 4–5). (M) Graphical abstract summarizing the study findings. Figure 8M was generated using https://www.biorender.com/. Data were presented as the mean ± SEM. Data from more than two groups were analyzed by one‐way ANOVA. *p < 0.05, **p < 0.01.

To explore the solution for improving pregnancy outcomes in aged mice via reversing the DM senescence, we designed the following intervention strategies by adoptive transfer of young BMDM and αIL‐6R (blocking IL‐6 receptors) alone or in combination, respectively (Figure 8A). The mice were divided into four groups: PBS group, YBMDM group, αIL‐6R group, and YBMDM+αIL‐6R group. Compared with the PBS group, both YBMDM and YBMDM+αIL‐6R groups exhibited marked reduced embryo resorption rates (Figure 8B,C) and increased maternal body weight at E11.5. However, the total number of embryos had no marked difference across all the groups (Figure S5A,B). To further determine whether the observed reduction in embryo resorption translated into improved perinatal outcomes, pregnant mice from each group were allowed to proceed to E18.5 and assessed for the number of fetuses, embryo resorption rate, mean fetal weight, and mean placental weight. Consistent with the reduced resorption rates observed at earlier gestational time points, YBMDM‐treated aged dams exhibited a significantly higher number of fetuses at E18.5 compared with PBS‐treated controls (Figure S5C,D). Notably, embryo resorption rate, mean weight of fetuses, and placentas were not significantly different across all groups (Figure S5E–G).

HE staining showed that the placental area and vascularity in the decidual layer of the placenta at E11.5 were markedly increased in the YBMDM, αIL‐6R, and YBMDM+αIL‐6R groups compared with the PBS group. Immunofluorescence staining revealed a marked increase in CD31 expression and large‐lumen vessels in the decidual layer of the placenta at E11.5 in the YBMDM and YBMDM+αIL‐6R groups compared with the PBS group (Figure 8D,E).

To further underly the potential explanation for the beneficial interventive efficacy, FCM was used to detect the proportions of P53^+^DM, polarized macrophage subsets, and cytokine production levels. Our results showed that the proportion of P53^+^ DM was markedly decreased in the YBMDM and the YBMDM+αIL‐6R groups compared with the PBS group, whereas no marked difference was observed in the αIL‐6R group (Figure 8F,G). No marked differences were observed in M2‐type macrophages or in the M1/M2 ratio across all groups (Figure 8H–K). The produced levels of TNF‐α were markedly decreased in the YBMDM+αIL‐6R group at E11.5 (Figure 8L) compared to the αIL‐6R group, whereas levels of IL‐6, IFN‐γ, TGF‐β, and IL‐10 remained unchanged among the groups (Figure S5H).

Taken together, these findings demonstrate that transfer of young BMDM is beneficial for improving the pregnant outcomes in AMA mice, which might be a potential intervention strategy.

## 4 Discussion

With the advancement of modern society, the increasing trend of delayed maternal age has led to a significant rise in the proportion of women with AMA. The decline in fertility and the elevated incidence of pregnancy complications associated with AMA have emerged as significant public health concerns (Jacobsson et al. 2004; Palatnik et al. 2020). Through an integrative approach involving clinical samples, animal models, and in vitro experiments, this study firstly uncovers the presence of DM senescence in AMA pregnancies. Phenotypically, senescent DM is characterized by an increased proportion of pro‐inflammatory M1‐type macrophages (CD86^+^CD206^−^), a decreased proportion of anti‐inflammatory M2‐type macrophages (CD86^−^CD206^+^), an elevated M1/M2 ratio, and upregulated expression of senescence markers (P53, ROS, and SA‐β‐Gal). Functionally, these macrophages exhibit impaired phagocytic capacity, enhanced secretion of the pro‐inflammatory cytokine TNF‐α, and inhibition of trophoblast cell migration, invasion, and proliferation. The senescence mechanism of DM involves two interconnected pathways: first, low expression of FOXO3, which suppresses mitophagy by downregulating the PINK1/PARKIN pathway; second, increased IL‐6 levels in the uterine microenvironment accelerate senescence through activation of the pSTAT3 signaling pathway. Notably, adoptive transfer of BMDM from young mice significantly reduces embryo resorption and promotes placental development in AMA mouse models (Figure 8M). These findings provide novel mechanistic insights and potential therapeutic targets for improving pregnancy outcomes in AMA.

In experiments with aged mice, we found that the increased risk of age‐related adverse pregnancy outcomes was associated with female mice (10–12 months old) rather than male mice (10–12 months old). Consistent with our findings, Xiong et al. (2021) also reported that litter size in mice was not associated with the age of male mice within the 10–12‐month range. However, a meta‐analysis of human studies suggested that the risk of miscarriage increases with paternal age: the estimated risk of miscarriage in men aged ≥ 45 years was slightly higher (1.74; 95% CI 1.26, 2.41), with men aged 25–29 years as the reference group. These findings collectively suggest that advanced reproductive aging may occur later in males than in females. Therefore, future studies investigating the relationship between advanced paternal age and adverse pregnancy outcomes should include gradient experiments using male mice of older ages, to clarify the threshold of paternal age that affects pregnancy outcomes.

Using FCM, we systematically analyzed the proportion of early pregnancy DM in young and aged human and mouse models. Our results revealed a significant reduction in DM proportion in the aged group compared to the young group, consistent with findings by Woods et al. (2017), who reported decreased DM and dendritic cells (DC) proportions in aged (12‐month‐old) mouse placentas at E11.5, while no change in uNK. Notably, a contrasting study observed increased endoneurial macrophages in 24‐month‐old C57BL/6 mice compared to 12‐month‐old ones, accompanied by nerve fiber demyelination, remyelination, and axonal injury (Yuan et al. 2018), which may stem from differences in mouse age and tissue‐specific macrophage populations analyzed. In enteric nerve research, Becker et al. (2018) demonstrated that aging reduced the mRNA expression levels of M2 markers (CD206, FIZZ1, TGF‐β) and increased IL‐6 mRNA levels in mouse intramuscular macrophages and BMDM. Similarly, Shen et al. (2024) reported an age‐related increase in ovarian M1 macrophages (CD86^+^) and decreases in M2 macrophages (CD163^+^) in 6‐ and 9‐month‐old mice compared with 3‐month‐old mice, aligning with our observation of diminished M2 polarization in aged early pregnancy DM. Collectively, these results indicate that, relative to the young group, the aged group exhibits a lower proportion of early pregnancy M2‐type DM and a more pronounced pro‐inflammatory phenotype. Notably, splenic macrophage polarization remained unaltered between the aged and young groups, likely due to their distinct ontogeny from DM. DM originate from gestational peripheral monocyte infiltration combined with indigenous uterine macrophages (Sun et al. 2021), whereas splenic macrophages stem from embryonic‐seeded progenitors (Yona et al. 2013).

Current challenges in studying macrophage senescence lie in the absence of standardized biomarkers. Based on a comprehensive literature review, key hallmarks of macrophage senescence include altered cellular morphology, enhanced pro‐inflammatory phenotypes, upregulated expression of senescence‐associated proteins (P16, P21, P53), increased SA‐β‐Gal activity, reduced phagocytosis, impaired autophagy, elevated SASP, and diminished tissue repair capacity (Sharma 2021). In this study, we found significantly higher proportions of P53‐positive cells in DM from aged human and mouse samples compared to young controls. This finding is consistent with the report by Li et al. (2022), which showed that the Bazi Kidney Tonic Formula alleviated macrophage senescence in the brain of aging mice by down‐regulating the mRNA levels of P53, Caspase‐3, and Bax, thereby supporting the utility of P53 as a marker of macrophage senescence.

To address the limitation of relying solely on P53, we also evaluated SA‐β‐Gal expression, a classic senescence marker. We found significantly higher SA‐β‐Gal positivity in DM and MDMs from aged individuals compared to young controls. These findings align with previous studies. For example, diabetes‐induced retinal macrophage senescence is characterized by increased SA‐β‐Gal expression, and α‐Klotho mitigates macrophage senescence and neovascular lesions by down‐regulating HECT domain containing 1 and reducing insulin receptor substrate 1 ubiquitination (Q. B. Li et al. 2024). Additionally, we observed elevated accumulation of ROS in aged DM of both humans and mice, a previously unreported finding that further validates DM senescence in AMA pregnancies. Together, these results confirm that DM in early pregnancy under AMA conditions exhibits clear senescence characteristics, including reduced cellularity, skewed M1/M2 polarization, and upregulated senescence markers (P53, ROS, SA‐β‐Gal).

Macrophages play pivotal roles in various stages of pregnancy, including decidualization, trophoblast invasion, angiogenesis, parturition, and postpartum uterine repair. Dysfunctional macrophages contribute to pregnancy complications, including miscarriage, PE, and preterm birth (Yao et al. 2019). Notably, macrophages are essential for clearing senescent cells in the postpartum uterus. In a mouse model, blocking macrophages by postnatal abdominal injection of anti‐F4/80 antibody expanded SA‐β‐Gal‐positive regions at placental implantation scar sites (Egashira et al. 2017), indicating impaired senescent cell clearance. Our study demonstrated that DM in both AMA pregnant women and mice exhibited significantly reduced phagocytic ability compared to their younger counterparts. This finding is consistent with research on aging during influenza infection, which reported that aging impairs alveolar macrophage phagocytosis. Specifically, it leads to decreased phagocytosis of apoptotic neutrophils by alveolar macrophages, down‐regulation of the clearance receptor CD204, increased lung injury, and a higher mortality rate in elderly individuals infected with the influenza virus (Wong et al. 2017). Similarly, Minhas et al. (2019) showed that senescent macrophages suppress mitochondrial NAD^+^‐dependent signaling, leading to diminished phagocytic function (Blacher et al. 2022). Consequently, the reduced DM phagocytic capacity in early pregnancy may hinder the removal of damaged and senescent cells in the uterus and placenta, posing a threat to pregnancy outcomes in AMA.

Macrophages contribute to maternal‐fetal immune tolerance by secreting cytokines to regulate immune responses. Our research revealed that early pregnancy DM from AMA mice produced elevated levels of the proinflammatory cytokines, such as TNF‐α and IL‐6, compared to the younger group. During pregnancy, increased TNF‐α levels can disrupt hormone synthesis, impede trophoblast cell migration, alter placental structure, and affect embryonic development, all of which are associated with pregnancy complications like miscarriage and PE (Romanowska‐Próchnicka et al. 2021; Toder et al. 2003). Zeng et al. (2023) found increased TNF‐α and TNFR1 expression in the decidua of women with recurrent pregnancy loss. This upregulation induced excessive senescence of decidual stromal cells through the increased expression of P53 and P16 proteins. Similarly, studies have found that compared with normal pregnancy, IL‐6 expression in decidual tissues of women with recurrent pregnancy loss was increased (Vilotic et al. 2022). These findings indicate that the enhanced secretion of TNF‐α and IL‐6 by early pregnancy DM in AMA may contribute to the development of pregnancy complications by negatively impacting embryonic and placental development.

A successful pregnancy depends on the normal development of both the embryo and placenta, with trophoblast cell differentiation and function playing a critical role (Horii et al. 2020; Knöfler et al. 2019). Our study showed that culture supernatants from early pregnancy MDMs of the aged group significantly suppressed the migration, invasion, and proliferation of trophoblasts compared to the young group. In early pregnancy, extravillous trophoblasts migrate and invade maternal spiral arteries to remodel them, ensuring sufficient nutrient and oxygen delivery to the fetus. Impaired extravillous trophoblast invasion has been linked to pregnancy complications, including PE and fetal growth restriction (Meakin et al. 2022; Moser et al. 2018). Thus, we propose that the inhibitory effect of DM from the aged group on trophoblast function may contribute to the increased incidence of adverse pregnancy outcomes in AMA pregnancies.

In conclusion, early pregnancy DM in AMA is characterized by diminished phagocytic capacity and enhanced secretion of proinflammatory cytokines. These changes disrupt the trophoblasts' biological function and are linked to a higher risk of adverse pregnancy outcomes.

Transcriptome sequencing of DM sorted by FACS from early pregnancy samples of young and aged humans revealed that DM senescence may be linked to reduced expression of FOXO3. As a longevity‐associated gene, FOXO3 participates in a range of biological processes, including inflammation, autophagy, glycolysis, DNA damage repair, and cell cycle arrest. It delays telomere shortening, promotes cell self‐renewal, and maintains genome stability, thereby decelerating the aging process and exerting anti‐aging effects (Cao et al. 2023; McIntyre et al. 2022). Single‐cell transcriptomic analyses of aortic and coronary artery tissues from young and aged cynomolgus macaques identified FOXO3 as a key regulator of vascular aging; its deletion accelerates arterial endothelial senescence (W. Q. Zhang et al. 2020). Similarly, in aged primates, FOXO3 is downregulated in skeletal muscle, where it acts as a crucial transcription factor for maintaining muscle homeostasis. FOXO3 knocking down in human myotubes hastens their aging, whereas its activation attenuates it (Jing et al. 2023). Becker et al. (2018) also reported that FOXO3 expression declines with age in mouse enteric neuromuscular macrophages and BMDM, consistent with our finding that *Foxo3* mRNA levels were significantly reduced in aged mice BMDM compared to those of young mice. These results further indicate the hypothesis that macrophages may undergo age‐related genetic modifications prior to entering the uterine microenvironment. Although both RNA‐seq and RT‐PCR data showed decreased *CREB5* in DM from AMA women, we did not verify its expression in aged mouse BMDMs. Further research is required to clarify whether CREB5 as FOXO3 contributes to macrophage senescence and compromised pregnancy outcomes in AMA.

Previous research showed that ethanol‐induced hepatic macrophages in wild‐type mice exhibited increased mRNA levels of *Tnf*, *Il6*, *Il1b*, and *Inos*, along with decreased *Arg1 mRNA* levels (Z. Li et al. 2018). Consistent with these findings, our study demonstrated that the FOXO3‐knockdown THP‐1 stably transduced strain showed significantly elevated INOS (an M1‐type macrophage marker) protein expression, as well as increased mRNA levels of *Tnf and Il6* detected by RT‐qPCR. Collectively, these data suggest that low FOXO3 expression can regulate DM senescence. However, the underlying regulatory mechanism remains unclear.

Mitochondria play a pivotal role in cellular senescence (López‐Otin et al. 2023), and mitophagy, which involves engulfing damaged mitochondria, is considered a longevity‐promoting mechanism. It operates through two primary pathways: PINK1/PARKIN‐dependent (ubiquitin‐dependent) and non‐PINK1/PARKIN‐dependent (ubiquitin‐independent) pathways (Picca et al. 2023; Zhu et al. 2021). Audesse et al. (2019) demonstrated that overexpressing FOXO3 in neural stem and progenitor cells upregulates PINK1, indicating that FOXO3 regulates mitophagy by transcriptionally controlling downstream PINK1. Our findings support hypothesis: FOXO3 knockdown in THP‐1‐derived macrophages (the KD group) showed reduced PINK1/PARKIN protein levels in the mitophagy signaling pathway. Transcriptomic analysis further revealed downregulation of the mitophagy pathway in the KD group relative to the negative control group. Therefore, we propose that FOXO3 deficiency inhibits mitophagy by suppressing the PINK1/PARKIN axis, leading to mitochondrial dysfunction and ultimately driving macrophage senescence.

Serum IL‐6 levels increase significantly with age and are linked to aging and chronic disease development. Higher IL‐6 correlates with reduced physical (as measured by the Daily Living Ability Scale) and cognitive performance (as measured by the Mini‐Mental State Examination) (Puzianowska‐Kuznicka et al. 2016). In this study, early pregnancy decidua from human aged groups showed significant senescence compared to young groups, including elevated expression of senescence markers P16, P21, and P53, as well as elevated proinflammatory cytokine IL‐6 expression. This is consistent with Xiong et al. (2021), who found reduced SIRT1 and increased P53/P21 expression in AMA early decidual and late placental tissues, indicating placental aging. However, our IHC analysis only detected global upregulation of senescence biomarkers in decidual tissues from AMA but could not attribute these signals to defined cell subsets, as cell‐specific co‐staining was not implemented in the current experimental design. Follow‐up experiments combining multiplex immunofluorescent co‐localization and single‐cell transcriptomics will help accurately characterize senescent cell subtypes in situ (Harms et al. 2023).

Notably, although the production of both IL‐6 and TNF‐α is increased in DMs from aged pregnancies, they occupy disparate tiers in our proposed pathogenic cascade. IL‐6 is widely induced across decidual tissues as a core SASP mediator that acts as an extrinsic microenvironmental stimulus to promote DM senescence, while TNF‐α mainly represents a terminal SASP factor secreted by senescent DM. Consistent with this division of roles, our functional neutralization experiments focused specifically on IL‐6‐dependent paracrine signaling. Future investigations are required to clarify whether TNF‐α impairs DM function alone or via synergistic crosstalk with IL‐6.

To explore whether IL‐6, as a key uterine microenvironment molecule in AMA pregnancy, accelerates DM senescence, we treated THP‐1‐derived macrophages with IL‐6 and observed increased INOS and P53 protein expression—aligning with Chen et al. (2022), who linked IL‐6/STAT3 pathway activation to increased M1 macrophages in *Porphyromonas gingivalis* ‐induced apical inflammatory milieu. Our study further showed IL‐6 increased pSTAT3 protein expression in the NC group, indicating that IL‐6 might induce macrophage senescence via the STAT3 signaling pathway. However, direct evidence proving pSTAT3 mediates IL‐6‐driven macrophage senescence remains absent; targeted STAT3 blockade will be required to validate this regulatory axis in subsequent studies.

Given that both genetic and environmental factors influence DM senescence, we performed rescue experiments for further confirmation. At the genetic level, DM senescence was reversed by adoptive transfer of BMDM from young mice, while at the environmental level, DM senescence was attenuated by injection of αIL‐6R. Previous studies showed that adoptive transfer of BMDM‐derived M2 macrophages (induced by IL‐4/IL‐13) at E15.6 and E16.5 could prevent preterm birth and improve neonatal survival in a LPS‐induced intraamniotic inflammation model (Gomez‐Lopez et al. 2021). Additionally, transfer of M2‐polarized macrophages derived from splenocytes of 6–8‐week‐old female BALB/c mice to the recipient BALB/c mice on E3.5, followed by an injection of LPS (0.5 μg/100 μL) on E6.5 to induce the abortion, significantly reduced the absorption rates. In contrast, transfer of M1 splenic macrophages had little to no effect (Y. Q. Liu et al. 2021). αIL‐6R, which blocks IL‐6 by binding its receptor, is widely used in rheumatoid arthritis (Barber et al. 2014), cancer (Hong et al. 2022), and acute psychological stress (Qing et al. 2020). IL‐6 blockade could restore damaged CD4^+^T cells and promote CD8^+^T cell‐dependent tumor elimination in old mice (Tsukamoto et al. 2015). Our findings demonstrate that young BMDM transfer could reduce the embryo resorption rates and improve the placental development in AMA mice, offering new intervention strategies for AMA‐related adverse pregnancy outcomes. Nevertheless, we did not track the in vivo engraftment of adoptively transferred BMDM within the recipient endometrium in the current study, thus cannot exclude indirect extrauterine effects of transferred BMDM on pregnancy outcomes. Future experiments utilizing CD45.1/CD45.2 congenic or GFP‐transgenic donor mice combined with flow cytometric profiling will enable precise quantification of donor‐derived BMDM abundance within the endometrium and other tissues.

In addition to DM, aged decidua harbors multiple cellular sources of IL‐6, including stromal cells, endothelial cells, T cells, and other immune cells (Yang et al. 2023). Senescent trophoblasts in AMA pregnancies also secrete IL‐6 as a key SASP factor (Z. Chen et al. 2021). These non‐DM‐derived IL‐6 sources might collectively shape a pro‐inflammatory and pro‐senescent maternal–fetal microenvironment, which may drive senescence and M1‐like polarization of adoptively transferred YBMDM within the aged endometrium. Future studies combining cell‐type‐specific *Il6* knockout models, single‐cell/spatial transcriptomics, and lineage tracing will help dissect the cellular origins of IL‐6 and clarify their specific roles in regulating YBMDM senescence in vivo.

Our study has several limitations that point to future directions for research. Firstly, although this study demonstrates that DM senescence in AMA impairs the biological functions of the trophoblasts, it does not rule out the possibility that DM senescence also affects the functions of other immune cells at the maternal‐fetal interface and decidual stromal cells. Secondly, primary decidual macrophage transcriptomics upon FOXO3 depletion from young women were not determined here due to restricted primary cell resources; future parallel RNA‐seq of edited primary DM and THP‐1 cells will facilitate direct cross‐comparison. Thirdly, neonatal sex was not recorded to enable subgroup birthweight analysis. In addition, spontaneous delivery leads to maternal consumption of placentae, resulting in unavailable placental weight data. Finally, embryo transfer causes unavoidable surgical stress, confounding the resorption rate in aged recipients. Thus, the high resorption rate in YE‐A mice (82.8%) might result from combined uterine aging defects and operative injury, and we cannot quantitatively distinguish their individual effects. Future refined surgical protocols will resolve this issue.

In summary, this study demonstrates that FOXO3 deficiency induces DM senescence via mitophagy impairment, while uterine IL‐6 accelerates senescence through STAT3 activation. Senescent DM disrupts trophoblast function and placental development, contributing to AMA‐associated adverse outcomes. Critically, adoptive transfer of young BMDM offer promising potential therapeutic avenues for intervention strategies. Future research should address challenges in donor cell acquisition and large‐scale clinical translation, while exploring small molecule activators of FOXO3 (e.g., octahydrocurcumin) (Calissi et al. 2021) and mitophagy (e.g., melatonin) (Luján et al. 2022) as alternative therapeutic approaches.

## Author Contributions

Y.Z. contributed to the concept, performed the experiments, analyzed the results, and wrote the manuscript. Y.Z., G.G., Z.L., and N.L. helped to perform the animal experiments. X.F., D.W., and Y.L. helped to perform the cell experiments. W.X. helped to perform the embryo transfer experiments. A.L. contributed to the concept, funding acquisition, manuscript revision, and final approval. All the authors have read the final version of the manuscript and agree to the submission.

## Funding

This study was supported by the grants from the National Natural Science Foundation of China (No. 82220108008), the Key R&D Programs of Hubei Province (No. 2022BAD088), and the Fundamental Research Funds for the Central Universities of China (No. YCJJ202201049).

## Ethics Statement

All animal procedures were conducted in accordance with the NIH Guide for the Care and Use of Laboratory Animals and were approved by the Institutional Animal Care and Use Committee (IACUC) of Huazhong University of Science and Technology (Wuhan, China; Protocol No. 4723[2022]). The human study was conducted in accordance with the Declaration of Helsinki and approved by the Clinical Trial Ethics Committee (CTEC) of Huazhong University of Science and Technology (Wuhan, China; Approval No. S154[2021]).

## Consent

Written informed consent was obtained from all participants.

## Conflicts of Interest

The authors declare no conflicts of interest.

## Supporting information


**Table S1:** Clinical characteristics.
**Table S2:** The lists of antibodies.
**Table S3:** Primer sequences and product sizes.
**Figure S1:** MOI and optimal infection conditions for lentivirus‐infected THP‐1 cells. (A) Representative brightfield images of THP‐1 cells treated with different concentrations of puromycin (0, 0.5, 1, 2, and 3 μg/mL) for 72 h. Scale bars = 40 and 20 μm. (*n* = 3) (B) Brightfield and fluorescence contrast images of THP‐1 cell infection by lentiviruses at different MOI (0, 1, 5, 10, and 30) without polybrene (5 μg/mL). Scale bar = 20 μm. (*n* = 3) (C) Brightfield and fluorescence contrast images of THP‐1 cell infection by lentiviruses at different MOI (0, 1, 5, 10, and 30) with polybrene (5 μg/mL). Scale bar = 20 μm. (*n* = 3) MOI: multiplicity of infection; Puro: Puromyci; Poly: Polybrene (gene transfection enhancer).
**Figure S2:** Generation and characterization of FOXO3 knockdown THP‐1 stable transgenic cells. (A) Representative brightfield and fluorescence microscopy images of THP‐1 cells infected with lentivirus at MOI = 10 and 5 μg/mL polybrene (*n* = 3). Scale bar = 20 μm. (B) RT‐qPCR analysis of FOXO3 mRNA expression levels in different groups (*n* = 3). (C, D) WB detection of FOXO3 protein expression levels in the Blank (control), NC (empty vector control), and KD (FOXO3 knockdown) groups (*n* = 3). Data were presented as the mean ± SEM. Statistical analysis: One‐way ANOVA for multi‐group comparisons. **p < 0.01, ***p < 0.001.
**Figure S3:** Comparison of decidual macrophage proportions and polarization in different mouse groups. FCM was used to quantify DM, M1, and M2 macrophage subsets, and M1/M2 ratios across mouse groups at E11.5 (YBMDM → Y group: *n* = 5; ABMDM → Y group: *n* = 5; YBMDM → A group: *n* = 4; ABMDM → A group: *n* = 5). Data were presented as the mean ± SEM. Multi‐group comparisons were performed using one‐way ANOVA.
**Figure S4:** In vitro verification of genetic and environmental factors jointly affecting placental development in mice. Representative placental HE staining images at E11.5 across different mouse groups. Scale bars = 500 and 50 μm. The blue and red frames indicate magnified regions of the decidua (Dec) and labyrinth (Lab) layers, respectively.
**Figure S5:** Comparison of pregnancy outcomes and decidual macrophages function in rescue experiments. (A) Comparison of embryo numbers at E11.5 across mouse groups (PBS group: *n* = 4; YBMDM group: *n* = 4; αIL‐6R group: *n* = 5; YBMDM+αIL‐6R group: *n* = 5). (B) Weight changes in mice from E0.5 to E11.5 across groups. (C, D) Comparison of number of fetuses at E18.5 across all groups (PBS group: *n* = 4; YBMDM group: *n* = 4; αIL‐6R group: *n* = 3; YBMDM+αIL‐6R group: *n* = 4). (E) Comparison of embryo resorption rates at E18.5 across all groups. (F, G) Mean weigh of fetuses and placentas at E18.5 across all groups. (H) FCM analysis of cytokine secretion (IL‐6, IFN‐γ, TGF‐β, IL‐10) by DM at E11.5 (PBS group: *n* = 5; YBMDM group: *n* = 4; αIL‐6R group: *n* = 5; YBMDM+αIL‐6R group: *n* = 5). Data were presented as the mean ± SEM. Multi‐group comparisons were performed using one‐way ANOVA.

## Data Availability

All data needed to evaluate the conclusions in the paper are present in the paper and/or the Supporting Information. Additional data that support the findings of this study are available from the corresponding author upon reasonable request.
