# Supplementary material for: Age‐Associated Senescence of Decidual Macrophages: A Key Mediator of Adverse Pregnancy Outcomes in Advanced Maternal Age
Source: Aging Cell. 2026 Jul 1;25(7):e70614. doi: 10.1111/acel.70614 (PMC13323841; doi:10.1111/acel.70614)
Supplement: Supplementary file 1 — Table S1: Clinical characteristics. Table S2: The lists of antibodies. Table S3: Primer sequences and product sizes. Figure S1: MOI and optimal infection conditions for lentivirus‐infected THP‐1 cells. (A) Representative brightfield images of THP‐1 cells treated with different concentrations of puromycin (0, 0.5, 1, 2, and 3 μg/mL) for 72 h. Scale bars = 40 and 20 μm. (n = 3) (B) Brightfield and fluorescence contrast images of THP‐1 cell infection by lentiviruses at different MOI (0, 1, 5, 10, and 30) without polybrene (5 μg/mL). Scale bar = 20 μm. (n = 3) (C) Brightfield and fluorescence contrast images of THP‐1 cell infection by lentiviruses at different MOI (0, 1, 5, 10, and 30) with polybrene (5 μg/mL). Scale bar = 20 μm. (n = 3) MOI: multiplicity of infection; Puro: Puromyci; Poly: Polybrene (gene transfection enhancer). Figure S2: Generation and characterization of FOXO3 knockdown THP‐1 stable transgenic cells. (A) Representative brightfield and fluorescence microscopy images of THP‐1 cells infected with lentivirus at MOI = 10 and 5 μg/mL polybrene (n = 3). Scale bar = 20 μm. (B) RT‐qPCR analysis of FOXO3 mRNA expression levels in different groups (n = 3). (C, D) WB detection of FOXO3 protein expression levels in the Blank (control), NC (empty vector control), and KD (FOXO3 knockdown) groups (n = 3). Data were presented as the mean ± SEM. Statistical analysis: One‐way ANOVA for multi‐group comparisons. **p < 0.01, ***p < 0.001. Figure S3: Comparison of decidual macrophage proportions and polarization in different mouse groups. FCM was used to quantify DM, M1, and M2 macrophage subsets, and M1/M2 ratios across mouse groups at E11.5 (YBMDM → Y group: n = 5; ABMDM → Y group: n = 5; YBMDM → A group: n = 4; ABMDM → A group: n = 5). Data were presented as the mean ± SEM. Multi‐group comparisons were performed using one‐way ANOVA. Figure S4: In vitro verification of genetic and environmental factors jointly affecting placental development in mice. Representative plac [file ACEL-25-e70614-s001.pdf]

Title: **Age-Associated Senescence of Decidual Macrophages: A Key Mediator of Adverse Pregnancy Outcomes in Advanced Maternal Age**

Authors: Yujing Zhang[1], Yiming Zhang[1], Guangshun Gong[1], Zhijing Li[1], Wenjing Xiong[2], Xuhui Fang[1], Ning Lu[1], Di Wang[1], Yihui Li[1], Aihua Liao[1,*]

Affiliations:

1. Institute of Reproductive Health, Center for Reproductive Medicine, Tongji Medical College, Huazhong University of Science and Technology, Wuhan, 430030, P.R. China.
2. Laboratory of Animal Center, Huazhong University of Science and Technology, Wuhan, 430030, P.R. China.

*Corresponding author: Ai-Hua Liao, MD, PhD

Institute of Reproductive Health, Center for Reproductive Medicine, Tongji Medical College, Huazhong University of Science and Technology, No.13 Hangkong Road, 430030 Wuhan, P.R China.

E-mail address: aihua_liao@hust.edu.cn

SUPPLEMENTAL INFORMATION

Supplementary Materials and Methods

Calculation of low-birth-weight fetuses

We defined fetuses with low-birth-weight (LBW) as those weighing >20% below the mean birth weight. This mean birth weight was calculated for each litter by first excluding the highest and lowest weight values (to eliminate the impact of extreme outliers) and then averaging the weights of the remaining pups. This cutoff was selected based on alignment with the relative deviation used for human LBW definitions and our experimental observations, where abnormal pups within the same litter consistently weighed >20% below the litter-specific average. The average birth weight used for this calculation was derived from all experimental litters (not exclusively Y×Y animals) to ensure consistency across groups.

Reactive oxygen species (ROS) Level Monitoring

Following surface antibody staining for flow cytometry (FCM), cell pellets were resuspended in 1 mL of serum-free RPMI 1640 medium. Next, 1 μL of 2′,7′-dichlorodihydrofluorescein diacetate (DCFH-DA; Beyotime, Cat. No. S0033S) was added, and the mixture was gently pipetted to ensure homogeneity. Cells were incubated at 37°C in a 5% CO_2 humidified incubator for 20 min, with gentle mixing every 5 min to prevent cell sedimentation. After incubation, samples were washed twice with phosphate-buffered saline (PBS) and resuspended in PBS. Intracellular ROS levels were immediately quantified using FCM.

SA-β-Gal Staining (Senescence-Associated β-Galactosidase Staining)

Cells cultured in 6-well plates were first washed twice with 1 mL of PBS per well after aspirating the culture medium. According to the manufacturer’s protocol for the SA-β-Gal Staining Kit (Beyotime, Cat. No. C0602), 1 mL of β-galactosidase fixation solution was added to each well, followed by incubation at room temperature for 15 min. Cells were then washed twice with PBS to remove residual fixative. Subsequently, 0.5 mL of β-galactosidase staining working solution was added to each well, and the plates were incubated overnight at 37°C in a CO_2-free incubator (to avoid pH fluctuations affecting

enzyme activity). After incubation, stained cells were visualized and imaged immediately using a bright-field optical microscope.

Phagocytosis assay

Following FCM antibody staining, human and mouse decidual cells were resuspended in 99 μL of RPMI 1640 complete medium. A 1 μL aliquot of the Latex Beads-Rabbit IgG-PE complex (Cayman Chemical, Cat. No. 600451) was added to the cell suspension, which was then gently mixed and incubated for 2 hours in a 37°C incubator with 5% CO_2. After incubation, cells were washed twice with PBS, resuspended in 200 μL of Assay Buffer (Cayman Chemical, Cat. No. 600451), and immediately analyzed via flow cytometry to quantify phagocytic capacity.

Migration assay

HTR-8/SVneo or SWAN71 cell lines were seeded into 12-well plates at a density of 5×105 cells/mL. Upon reaching ≥90% confluency, three vertical scratches were made on the well bottom using a 200 μL pipette tip with consistent pressure. The samples to be tested were added and photographed at the 0, 12, 24, 36, and 48 hours under a 4× magnification, ensuring consistent positioning for each photograph.

Invasion assay

Briefly, 70 μL of diluted Metrigel (1 mg/mL, BD, 354230) was added to the transwell upper compartment as previously described by our group (Xu et al., 2023). The Matrigel was incubated at 37 °C in a 5% CO_2 incubator for 4-5 hours to solidify. Subsequently, 1×105 HTR-8/SVneo or SWAN71 cells in serum-free RPMI-1640 medium were seeded into the upper chamber. 600 μL of the cell culture supernatant to be added in the lower chamber and incubated for 24 or 48 hours. The transwell chamber was removed and placed in a well containing 800 μL methanol, and fixed for 30 min at room temperature. The cells were washed twice with PBS, and the chambers were placed in wells of 800 μL of 0.1% crystal violet solution for 30 min at room temperature. A cotton swab was used to gently wipe the upper chamber bottom surface to remove cells and residual Matrigel, followed by immediate observation and imaging under a light microscope at 4× magnification.

Proliferation assay

HTR-8/SVneo or SWAN71 cells were seeded onto cell culture slides and cultured for 12 hours. Cell proliferation was evaluated using the EdU555 proliferation assay kit (Beyotime, cat. no. C0075S) following the manufacturer's protocol. After the initial 12-hour culture, cell culture supernatants were aspirated and replaced with fresh medium, and cells were further incubated for 24 hours. To label proliferating cells, 5-ethynyl-2′-deoxyuridine (EdU) was added to the medium to a final concentration of 5 μM, followed by a 6-hour incubation.

Subsequently, cells were gently washed twice with PBS to remove excess EdU. For permeabilization, 100 μL of 0.3% Triton X-100 was added to each slide, and cells were incubated at 4°C for 20 minutes. After permeabilization, slides were incubated with 100 μL of click reaction solution (prepared per the manufacturer's instructions) for 30 minutes at room temperature in the dark to visualize EdU-labeled cells. Nuclei were counterstained with 200 μL of DAPI (Servicebio, cat. no. G1012) for nuclear localization.

Slides were mounted using an anti-fluorescence quenching medium and sealed with transparent nail polish. EdU-positive (proliferating) cells were imaged immediately using a fluorescence microscope (ZEISS, Apotome3).

Isolation and culture of human peripheral blood mononuclear cells (PBMCs)

A total of 10 mL peripheral blood collected in heparin anticoagulation tubes was transferred with a pasteur pipette into a 50 mL centrifuge tube containing 10 mL of PBS under a biosafety cabinet. PBMCs were isolated by density-gradient centrifugation using Ficoll (TBD, LTS1077). Peripheral blood monocytes were sorted by magnetic-activated cell sorting (MACS) with $CD14^+$ microbeads (Miltenyi Biotec, 130-050-201). Cells were resuspended in complete RPMI-1640 medium and 50 ng/mL recombinant human macrophage colony stimulating factor (M-CSF) to induce differentiation. The cells were placed in a 37°C, 5% CO_2 incubator for 7 days, during which half-volume medium replacement was performed on days 3-4, and MDMs were harvested on day 7.

Extraction of Bone Marrow-Derived Macrophages（BMDMs）

Mice were euthanized, and whole bodies were immersed in 75% ethanol for 3–5 minutes

for surface disinfection. Hindlimbs were excised and transferred to PBS, followed by careful dissection to isolate femurs. Femurs were gently crushed using a mortar, and the resulting homogenate was filtered through a 200-mesh cell strainer to collect the supernatant. The supernatant was centrifuged at 450 × g for 10 minutes at 4°C. The cell pellet was resuspended in red blood cell (RBC) lysis buffer for 30 seconds to eliminate erythrocytes, then centrifuged again to recover the remaining cell pellet, which was subsequently counted using a hemocytometer.

Cells were seeded into 10 cm culture dishes at a density of 2×10^7 cells per dish, and 10 mL of high-glucose Dulbecco's Modified Eagle Medium (DMEM) complete medium supplemented with 20% L929 cell culture supernatant (as a source of macrophage colony-stimulating factor, M-CSF) was added. Cultures were maintained in a 37°C incubator with 5% CO_2. On day 3 of culture, 3–4 mL of fresh complete medium was added to replenish nutrients. Fully differentiated BMDMs were harvested for subsequent experiments on day 5.

Western blotting

Cells and triturated tissue samples were lysed on ice using RIPA lysate for 30 min. Protein extracts were determined by BCA assay. Equal amounts of protein were mixed with loading buffer, and denatured at 98 °C in a thermal block for 10 minutes. SDS-PAGE gels were prepared at corresponding concentrations based on the size of the target protein. Routine electrophoresis, proteins were transferred to membranes, blocked with 5% skim milk for 1 hour at room temperature rocking, incubated with primary antibodies overnight at 4 °C with shaking, and probed with secondary antibodies for 1 hour at room temperature with shaking. Protein bands were visualized and grayscale intensities were quantified using ImageJ software.. The dilution ratios of primary and secondary antibodies follow the instructions, and the antibodies used are listed in **Table S2**.

RNA Sequencing and Bioinformatic Analysis

DM sorted by FACS were fully lysed in 1 mL TRIzol and sent to Novogene for sequencing on dry ice. RNA integrity was assessed using an Agilent 2100 bioanalyzer for quality control of the extracted RNA. After library construction, the libraries were quantified and evaluated

using a Qubit 2.0 Fluorometer and the Agilent 2100 bioanalyzer to ensure library quality. Quantitative analysis of gene expression levels was performed separately for each sample, and correlation analysis of gene expression levels between samples was performed to select samples with $R^2 > 0.8$. We performed differential analysis of gene expression with the screening criteria $|\log2(\text{FoldChange})| \geq 1$ & $\text{padj} \leq 0.05$. Functional enrichment analysis of DEGs was performed using cluster Profiler software for GO terms and KEGG pathways, and protein–protein interaction analysis was conducted using the STRING database. Meanwhile, GSEA was performed based on GO and KEGG annotations, respectively.

Histology, Immunofluorescence (IF), and Immunohistochemistry (IHC) staining

Tissue specimens were trimmed to ≤5 mm in size and fixed in 4% paraformaldehyde for 24–48 hours. Following paraffin embedding, sections were processed through dewaxing, hydration, antigen retrieval, and serum blocking steps. For both IF and IHC, sections were incubated with primary antibodies overnight at 4°C in a humidified chamber, followed by incubation with secondary antibodies at room temperature for 40 minutes (protected from light for IF).

For IF staining, after secondary antibody incubation, nuclei were counterstained with DAPI. Slides were mounted using an anti-fluorescence quenching medium and sealed with nail polish. Fluorescence images were acquired immediately using a fluorescence microscope.

For IHC staining, after antigen retrieval, sections were treated with 3% hydrogen peroxide at room temperature for 10 minutes to block endogenous peroxidase activity. Following secondary antibody incubation, sections were developed with 3,3′-diaminobenzidine (DAB; MXB, cat. no. DAB0031), counterstained with hematoxylin to visualize nuclei, and sealed with neutral balsam. The specific antibodies used in these experiments are listed in Table S2.

Establishment and Validation of FOXO3 Knockdown THP-1 cell lines

Lentivirus packaging was conducted by Jiman Bio Intro (China). A pilot experiment was first performed to optimize lentiviral infection conditions, confirming a multiplicity of

infection (MOI) of 10 with the addition of 5 μg/mL polybrene as optimal. Puromycin was used for selection at a concentration of 1 μg/mL and for long-term maintenance at 0.5 μg/mL. THP-1 cells were infected with lentivirus for 24 hours, after which the medium was replaced and cells were cultured for an additional 72 hours. When the infection efficiency exceeded 80%, cell suspensions were collected into 1.5 mL microcentrifuge tubes, centrifuged at 2000 rpm for 2 minutes, and the supernatant was discarded. Next, cells were resuspended in 1 mL of fresh medium supplemented with 1 μg/mL puromycin; the antibiotic-containing medium was refreshed every 3 days until all uninfected cells were eliminated. Thereafter, the established cell lines were maintained in medium with 0.5 μg/mL puromycin. The efficiency of FOXO3 knockdown was validated using western blotting and RT-qPCR.

Mito-Tracker and Mito-SOX staining

Mito-Tracker Deep Red 633 (Beyotime, cat. no. C1034; 1 μL) and Mito-SOX (Beyotime, cat. no. S0061S; 2 μL) were prewarmed to 37°C and mixed with 2 mL of complete RPMI-1640 medium to prepare the staining solution. The cell culture supernatant was carefully aspirated, and 2 mL of the staining solution was added to the cells, followed by incubation at 37°C for 20 minutes. Next, 1 μL of Hoechst 33342 live-cell nuclear staining reagent was added, and the cells were incubated at 37°C for 10 minutes in the dark. After incubation, the cells were washed twice with 2 mL of fresh complete RPMI-1640 medium each time and immediately imaged using an LSM900 confocal microscope.

Table S1. Clinical characteristics

	**20-29 y (n = 17)**	**30-34 y (n = 15)**	**≥35 y (n = 13）**	***P*-value**
Age (y)	24 (21, 29)	32 (30, 34)	39 (35, 41)	＜0.05
Gestational Age (day)	47 (43, 71)	48 (36, 68)	48 (40, 79)	＞0.05
Gravidity (number)	1 (0, 4)	2 (0, 4)	2 (1, 8) *	＜0.05
Parity (number)	0 (0, 2)	1 (0, 2)	1 (0, 2)	＞0.05

Data were presented as the median (maximum, minimum). Multi-group comparisons were performed using one-way ANOVA. *≥35y vs. 20-29y: $P < 0.05$.

Table S2. The lists of antibodies

Name	Cat	Source
HRP conjugated Goat Anti-Rabbit IgG	GB23303	Servicebio
HRP conjugated Goat Anti-Mouse IgG	GB23301	Servicebio
594-conjugated Goat Anti-Rabbit IgG	SA00013-4	Proteintech
488-conjugated Goat Anti-Mouse IgG	SA00013-1	Proteintech
Fluorescein (FITC)-conjugated Donkey Anti-Goat IgG	SA00003-3	Proteintech
594-conjugated Donkey Anti- Rabbit IgG	34212ES60	Yeasen
P16 antibody	10883-1-AP	Proteintech
P21 antibody	10442-1-AP	Proteintech
P53 antibody	28248-1-AP	Proteintech
SDHA antibody	SC-166909	Santa Cruz
FOXO3 antibody	10849-1-AP	Proteintech
CD68 antibody	SC-17832	Santa Cruz
PINK1 antibody	23274-1-AP	Proteintech
PARKIN antibody	66674-1-Ig	Proteintech
PE/Cyanine7 anti-human CD206	2450616	Invitrogen
PE anti-human CD86	2696748	Invitrogen
FITC anti-human CD14	308104	BioLegend
APC anti-human TNF-α	502912	BioLegend
APC anti-human IL-10	506807	BioLegend
APC anti-human IFN-γ	502512	BioLegend
APC anti-human TGF-β1	300006	BioLegend
PerCP/Cyanine5.5 anti-human IL-6	501118	BioLegend
FITC anti-mouse F4/80	B377381	BioLegend
PE anti-mouse F4/80	123110	BioLegend
PE anti-mouse CD86	105008	BioLegend
PerCP/Cyanine5.5 anti-mouse CD206	141716	BioLegend
PE anti-mouse IL-4	504103	BioLegend
PE/Cyanine7 anti-mouse IL-17A	2550884	Invitrogen
APC anti-mouse CD3	100236	BioLegend
FITC anti-mouse NK1.1	156508	BioLegend
APC/Cyanine7 anti-mouse CD45	103116	BioLegend

Name	Cat	Source
FITC anti-mouse CD4	100510	BioLegend
PE anti-mouse CD25	102007	BioLegend
APC anti-mouse IL-6	504508	BioLegend
PerCP/Cyanine5.5 anti-mouse IL-10	505028	BioLegend
PE/Cyanine7 anti-mouse TNF-α	506324	BioLegend
APC anti-mouse IFN-γ	505810	BioLegend
APC anti-mouse TGF-β	2721450	Invitrogen
P16 AF647 Antibody	SC-1661- AF647	Santa Cruz
P21 AF647 Antibody	SC-6246-AF647	Santa Cruz
P53 AF647 Antibody	SC-126-AF647	Santa Cruz

Table S3. Primer sequences and product sizes

Name	Forward primer	Reverse primer
Human *IL6*	AGCCACTCACCTCTTCAGAAC	GCAAGTCTCCTCATTGAATCCAG
Human *IL8*	CTGTGTGAAGGTGCAGTTTTGCC	CGCAGTGTGGTCCACTCTCAATC
Human *IFNG*	GAGTGTGGAGACCATCAAGGAAG	TGCTTTGCGTTGGACATTCAAGTC
Human *TNF*	CTCTTCTGCCTGCTGCACTTTG	ATGGGCTACAGGCTTGTCACTC
Human *IL1B*	CCACAGACCTTCCAGGAGAATG	GTGCAGTTCAGTGATCGTACAGG
Human *IL1A*	AAGATGAAGACCAACCAGTGC	AACAAGTTTGGATGGGCAACT
Human *CCL2*	CAGCCAGATGCAATCAATGCC	TGGAATCCTGAACCCACTTCT
Human *CREB5*	CCCTGCCCAACCCTACAATG	GGACCTTGCATCCCCATGAT
Human *NFKB1*	CATATTTGGGAAGGCCTGAACA	CCCACATAGTTGCAGATTTTGAC
Human *EIF4E*	ATGCCTGGCTGTGACTACTCAC	GAGGTCACTTCGTCTCTGCTGT
Human *P16*	CCAACGCACCGAATAGTTACG	GCGCTGCCCATCATCATG
Human *P21*	GACAGCAGAGGAAGACCATGTGGAC	GAGTGGTAGAAATCTGTCATGCTG
Human *P53*	GAGCTGAATGAGGCCTTGGA	CTGAGTCAGGCCCTTCTGTCTT
Human *FOXO3*	TCTGAACTCCCTACGCCAGT	GAGTCCGAAGTGAGCAGGTC
Human *β-ACTIN*	CATGTACGTTGCTATCCAGGC	CTCCTTAATGTCACGCACGAT
Mouse *p16*	CCCAACGCCCCGAACT	GCAGAAGAGCTGCTACGTGAA
Mouse *p21*	GTCAGGCTGGTCTGCCTCCG	CGGTCCCGTGGACAGTGAGCAG
Mouse *p53*	CATCACCTCACTGCATGGAC	TGAGGGGAGGAGAGTACGTG
Mouse *Il1b*	GCAACTGTTCCTGAACTCAACT	ATCTTTTGGGGTCCGTCAACT
Mouse *Tnf*	TCTTCTCATTCCTGCTTGTGG	GGTCTGGGCCATAGAACTGA
Mouse *Il10*	AGGCGCTGTCATCGATTTCTC	GACACCTTGGTCTTGGAGCTTAT
Mouse *Foxo3*	TACGAGTGGATGGTGCGCTGT	TCATTCTGAACGCGCATGAAGC
Mouse *β-actin*	GGCTGTATTCCCCTCCATCG	CCAGTTGGTAACAATGCCATGT

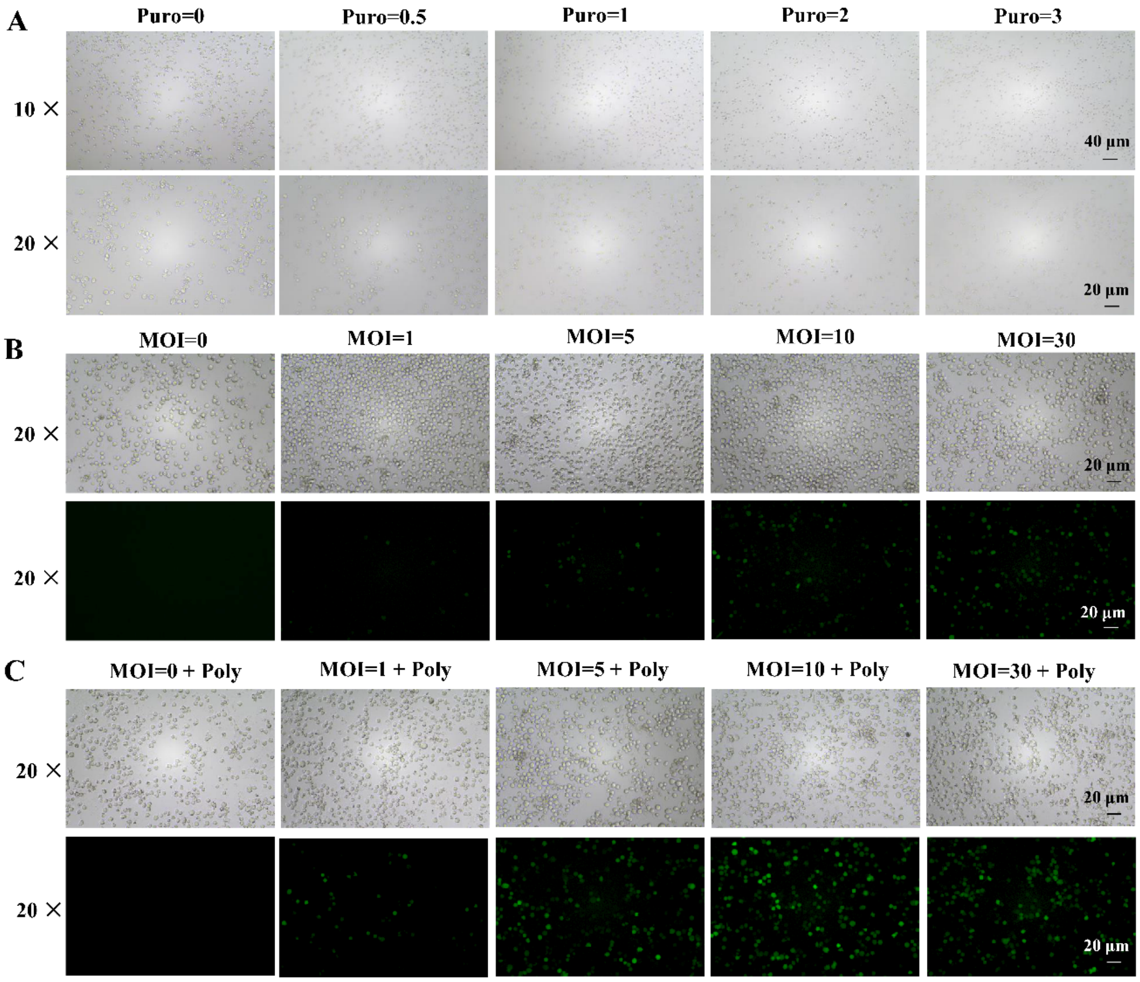

Figure S1. MOI and optimal infection conditions for lentivirus-infected THP-1 cells. (**A**) Representative brightfield images of THP-1 cells treated with different concentrations of puromycin (0, 0.5, 1, 2, and 3 μg/mL) for 72 hours. Scale bars = 40 μm and 20 μm. (*n* = 3) (**B**) Brightfield and fluorescence contrast images of THP-1 cell infection by lentiviruses at different MOI (0, 1, 5, 10, and 30) without polybrene (5 μg/mL). Scale bar = 20 μm. (*n* = 3) (**C**) Brightfield and fluorescence contrast images of THP-1 cell infection by lentiviruses at different MOI (0, 1, 5, 10, and 30) with polybrene (5 μg/mL).Scale bar = 20 μm. (*n* = 3) MOI: multiplicity of infection; Puro: Puromyci; Poly: Polybrene (gene transfection enhancer).

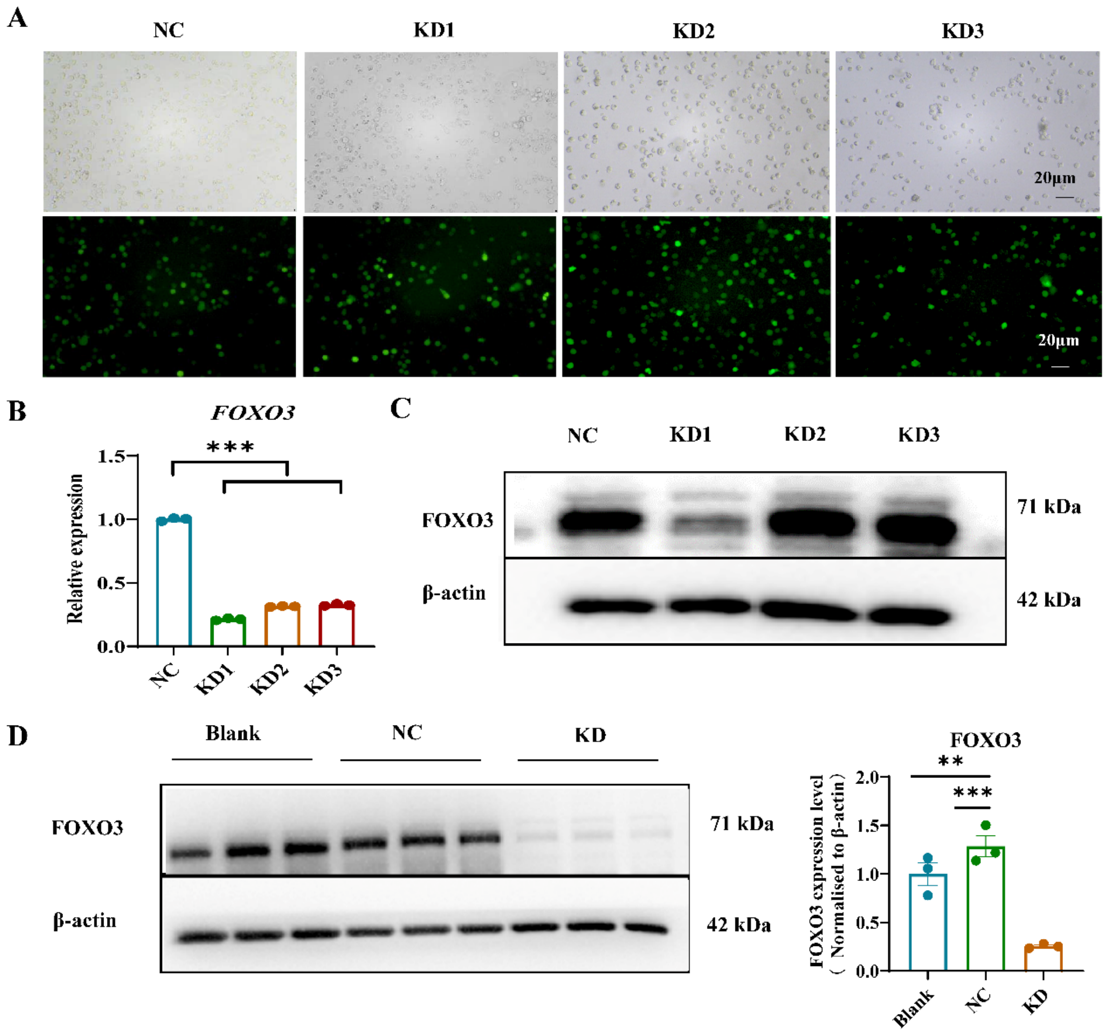

Figure S2. Generation and characterization of FOXO3 knockdown THP-1 stable transgenic cells. (**A**) Representative brightfield and fluorescence microscopy images of THP-1 cells infected with lentivirus at MOI = 10 and 5 μg/mL polybrene (*n* = 3). Scale bar = 20 μm. (**B**) RT-qPCR analysis of *FOXO3* mRNA expression levels in different groups (*n* = 3). (**C**, **D**) WB detection of FOXO3 protein expression levels in the Blank (control), NC (empty vector control), and KD (FOXO3 knockdown) groups (*n* = 3). Data were presented as the mean ± SEM. Statistical analysis: One-way ANOVA for multi-group comparisons. ** $P < 0.01$, *** $P < 0.001$.

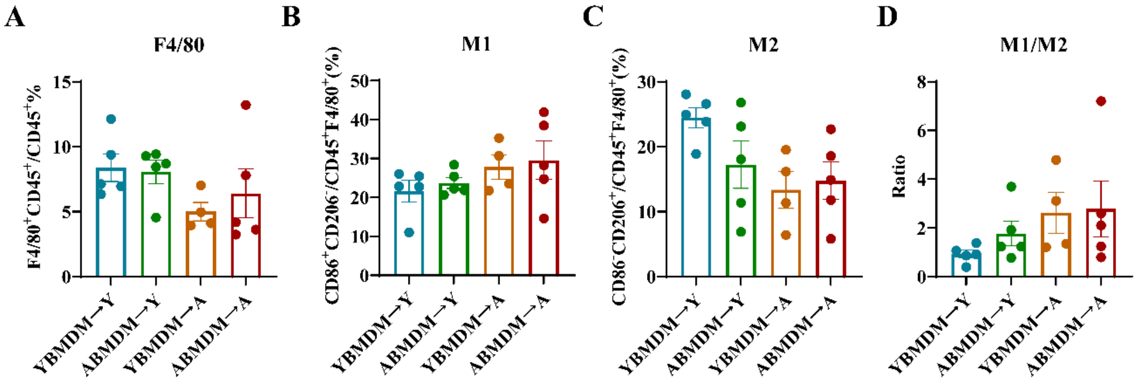

Figure S3. Comparison of decidual macrophage proportions and polarization in different mouse groups. FCM was used to quantify DM, M1 and M2 macrophage subsets, and M1/M2 ratios across mouse groups at E11.5 (YBMDM→Y group: $n = 5$; ABMDM→Y group: $n = 5$; YBMDM→A group: $n = 4$; ABMDM→A group: $n = 5$). Data were presented as the mean ± SEM. Multi-group comparisons were performed using one-way ANOVA.

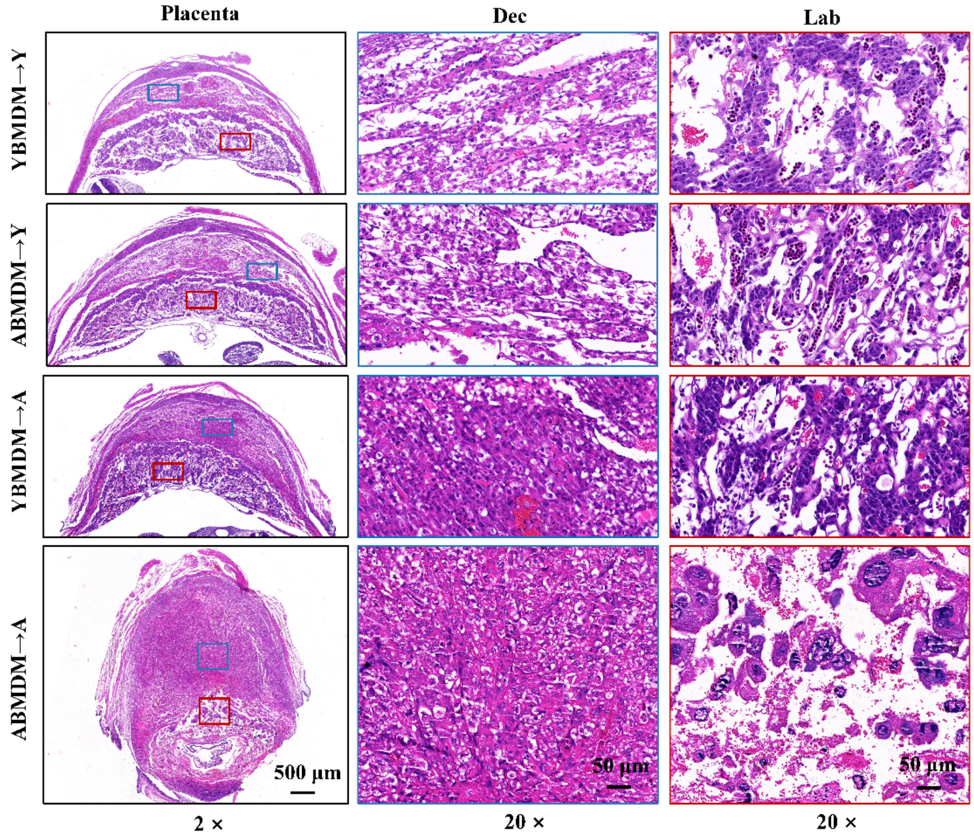

Figure S4. In vitro verification of genetic and environmental factors jointly affecting placental development in mice. Representative placental HE staining images at E11.5 across different mouse groups. Scale bars = 500 μm and 50 μm. The blue and red frames indicate magnified regions of the decidua (Dec) and labyrinth (Lab) layers, respectively.

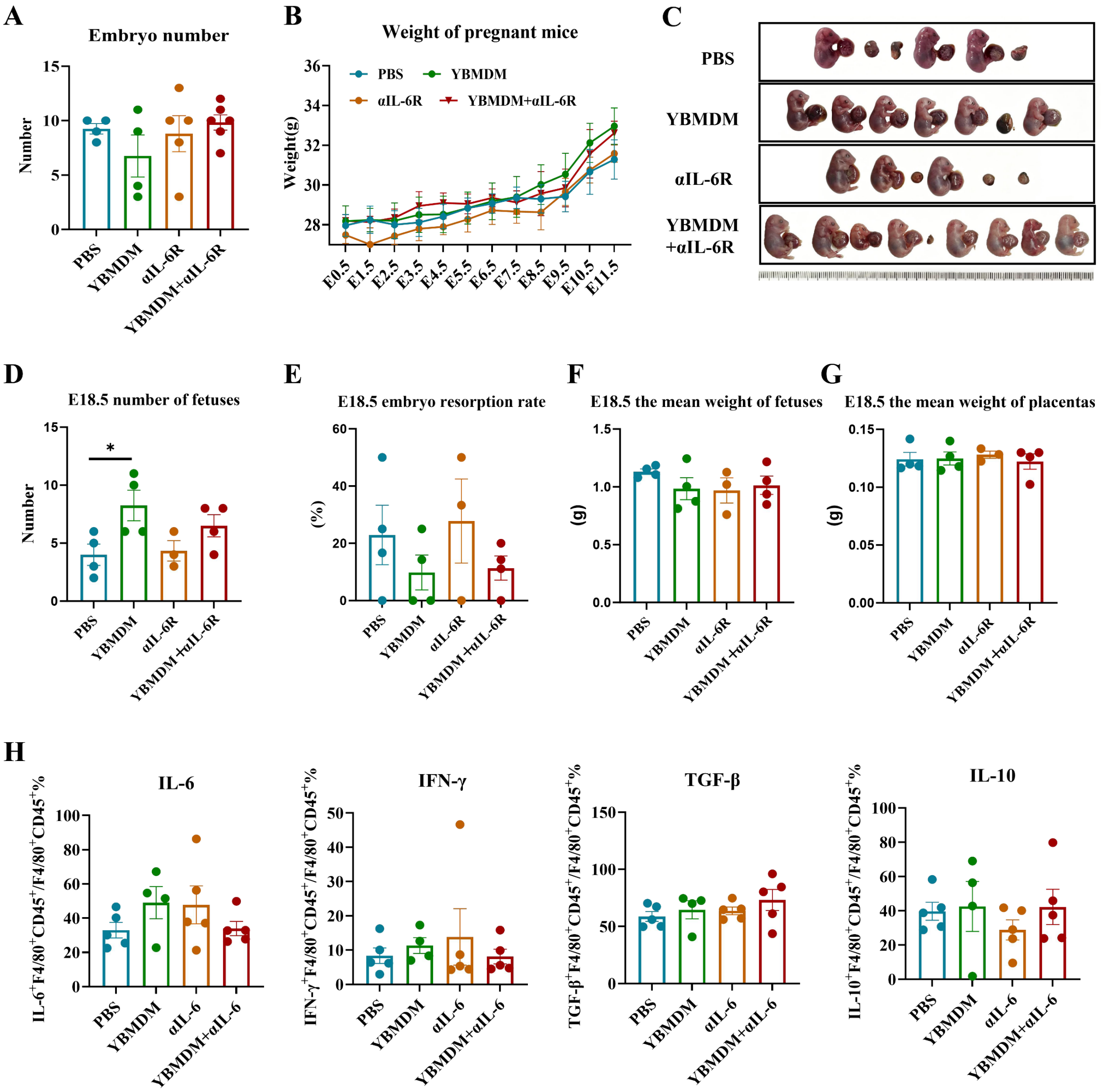

Figure S5. Comparison of pregnancy outcomes and decidual macrophages function in rescue experiments. (**A**) Comparison of embryo numbers at E11.5 across mouse groups (PBS group: *n* = 4; YBMDM group: *n* = 4; αIL-6R group: *n* = 5; YBMDM＋αIL-6R group: *n* = 5). (**B**) Weight changes in mice from E0.5 to E11.5 across groups. (**C,D**) Comparison of number of fetuses at E18.5 across all groups (PBS group: n = 4; YBMDM group: n = 4; α IL-6R group: n = 3; YBMDM＋α IL-6R group: n = 4). (**E**) Comparison of embryo resorption rates at E18.5 across all groups. (**F, G**) Mean weigh of fetuses and placentas at

E18.5 across all groups. **(H)** FCM analysis of cytokine secretion (IL-6, IFN-γ, TGF-β, IL-10) by DM at E11.5 (PBS group: *n* = 5; YBMDM group: *n* = 4; αIL-6R group: *n* = 5; YBMDM＋αIL-6R group: *n* = 5). Data were presented as the mean ± SEM. Multi-group comparisons were performed using one-way ANOVA.

Reference

Xu, Q. H., Muyayalo, K. P., Zhang, Y. J., Wang, H., Lin, X. X., & Liao, A. H. (2023). Altered vitamin D metabolism is involved in the dysregulation of γδT cell function and their crosstalk with trophoblasts in recurrent pregnancy loss. *American Journal of Reproductive Immunology, 89*(6). doi:ARTN e13581 10.1111/aji.13581
